# Speciation in the Peninsular Indian Flying Lizard (*Draco dussumieri*) Follows Climatic Transition and Not Physical Barriers

**DOI:** 10.1111/mec.17800

**Published:** 2025-05-20

**Authors:** Ramamoorthi Chaitanya, Aranya Dhibar, Akshay Khandekar, Channakesava Murthy, Shai Meiri, Praveen Karanth

**Affiliations:** ^1^ School of Zoology Tel Aviv University Tel Aviv Israel; ^2^ Centre for Ecological Sciences Indian Institute of Science Bangalore India; ^3^ Department of Integrative Biology University of Texas at Austin Austin Texas USA; ^4^ Thackeray Wildlife Foundation Mumbai India; ^5^ Department of Zoology Shivaji University Kolhapur India; ^6^ Zoological Survey of India, Western Ghats Regional Station Kozhikode India; ^7^ No. 86, N.G.O colony, Rajendra Nagar Mysore India; ^8^ The Steinhardt Museum of Natural History Tel Aviv University Tel Aviv Israel

**Keywords:** contact zone, isolation by environment, paleoclimate, population demographics, squamate, Western Ghats

## Abstract

Marked with high levels of endemism and in situ radiations, the Western Ghats mountains make for a compelling backdrop to examine processes that lead to the formation and maintenance of species. Regional geographic barriers and paleoclimatic fluctuations have been implicated as drivers of speciation, but their roles have not been explicitly tested in a phylogenomic framework. We integrated mitochondrial DNA, genome‐wide SNPs and climatic data to examine the influence of geographic barriers and climatic transitions in shaping phylogeography and potential speciation in the Peninsular Indian Flying lizard ( *Draco dussumieri* ). We found strong evidence for two independently evolving, geographically distinct, northern and southern lineages within *D. dussumieri* that diverged during the early Pleistocene, and a gradient of admixed populations across a broad hybrid zone in the Central Western Ghats. Migrations after initial divergence were continuous, but gene flow remained consistently below thresholds required to homogenise lineages. We found more support for isolation by environment (especially rainfall regimes) than by distance. The range‐break between lineages occurs at a transition zone in the Central Western Ghats that separates dissimilar rainfall regimes with no physical barriers. This limit is potentially an ecological barrier, which nevertheless was permeable during glacial maxima. We hypothesise that similar phylogeographic patterns will emerge in other widespread, wet‐adapted species in the Western Ghats that presumably endured the same climatic processes.

## 1 Introduction

Complex landscapes can often induce geographic and ecological isolation between populations, promoting substantial evolutionary differentiation (Brauer et al. 2018; Nali et al. 2020). Allopatric speciation can occur when closely related, ecologically similar populations separated by a physical barrier to gene flow accrue genetic differences over time that eventually lead to significant divergence (Wiens 2004; Pyron and Burbrink 2010). In continuous habitats, however, such populations tend to follow a pattern of isolation by distance (IBD), where genetic divergence is proportional to geographic distance (Wright 1943). Both forms of geographic isolation (by barrier and distance) induce population structure composed of divergence that is largely neutral (Baptestini et al. 2013). On the other hand, populations adapted to dissimilar local environments may experience differential ecological selection, leading to adaptive divergence (isolation by adaptation or environment; Nosil et al. 2008; Wang and Bradburd 2014). In complex landscapes, geographic and ecological isolating mechanisms can act in concert, posing challenges in detecting the causal factor that led to diversification (Mayr 1963; Coyne and Orr 2004).

Further, heterogeneous landscapes interacting with oscillating climatic cycles can facilitate gene flow between isolated populations, forming transient contact or hybrid zones in regions of sympatry. These zones serve as natural laboratories for studying reproductive isolation, as interactions between populations can either diminish or strengthen divergence (Johannesson et al. 2020). When reproductive isolation is incomplete, unrestricted gene flow can homogenise previously divergent lineages into a single, genetically admixed species. Conversely, if selection against hybrids is at play promoting assortative mating, gene flow may be limited, augmenting species boundaries.

Several patterns of introgression can emerge between admixing populations. For instance, if they are differentiated under ecological selection, adaptively divergent regions in the genome (‘genomic islands of divergence’) may be relatively better protected from homogenising gene flow than selectively neutral loci are (Barton and Bengtsson 1986). Alternatively, admixture may lead to adaptive introgression that can homogenise ‘genomic islands’ in populations, improving their ability to respond to local environmental change (Hedrick 2013; Song et al. 2011; Norris et al. 2015). Therefore, divergent populations that are in contact via pulsed or continuous migrations serve as excellent models for studying adaptive evolution and speciation in context of their spatiotemporal setting.

**FIGURE 1:**
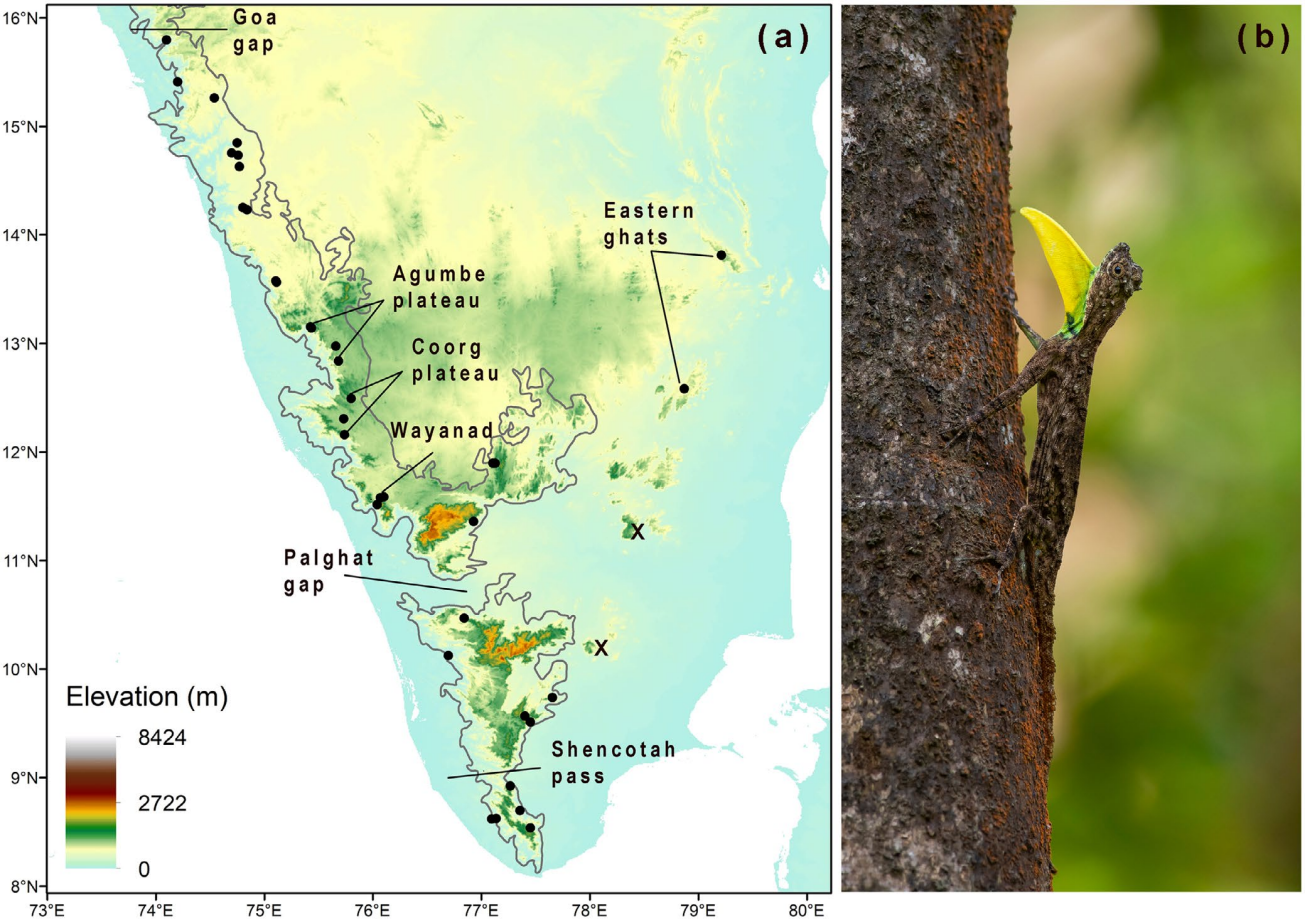
Elevation map of Peninsular India (a) with locations of *Draco dussumieri* sampled during this study. The contours of the Western Ghats are indicated by a solid line. Important regions and biogeographic barriers mentioned in the text are indicated. Hills in the southern Eastern Ghats marked with an ‘X’ refer to locations where *D. dussumieri* were found, but not collected (see Section 2). An adult male *D. dussumieri* (b) from the Agumbe plateau flashing his dewlap (Photo credit: Vinod Venugopal).

Peninsular India is an ecologically complex, geographically diverse landscape that endured severe climatic oscillations during the Pleistocene glacial cycles (2.58 Ma—11.7 ka; Joshi 1985; Srivastava et al. 2016). Monsoonal rainfall weakened during cooler periods of glacial expansion and intensified during warmer glacial recessions, and the intensity of these cycles was magnified during the Late Pleistocene (< 130 ka; see Saraswat et al. 2014 and the references therein). With its unique assemblage of biota, this landscape has been the setting for several phylogenetic and phylogeographic studies on vertebrates (Robin et al. 2010; Van Bocxlaer et al. 2012; Biju et al. 2011; Biju, Garg, Gururaja, et al. 2014; Biju, Garg, Mahony, et al. 2014; Vijayakumar et al. 2014; Robin et al. 2015; Agarwal and Karanth 2015; Pal et al. 2018; Chaitanya et al. 2019; Agarwal et al. 2019; Mallik et al. 2020, 2021 etc.) and invertebrates (Joshi and Edgecombe 2018; Joshi et al. 2020; Saha et al. 2022; Chakraborthy et al. 2024 etc.). The region is characterised by the largely wet forests of the Western Ghats and Eastern Ghats mountains that are separated by intervening arid lowlands (Figure 1a). While the Western Ghats are a continuous chain of mountains, interrupted only by the dry Palghat and Shencottah valleys in the south, the Eastern Ghats are composed of a set of discontinuous, smaller hill ranges. Both mountain systems harbour a gradient of moist deciduous to wet evergreen habitats at mid–high elevations that serve as the only refugia for wet‐adapted species in the region (Mani 1974; Biswas and Karanth 2021). Consequently, studies on several wet‐adapted taxa have shown cladogeneses that are spatially concordant with these geographic barriers (see Biswas and Karanth 2021 and the references therein). While these studies reported important phylogenetic patterns and quantified divergence between candidate lineages across biogeographic gaps, they did not estimate demographic histories or gene flow between closely related lineages (but see Khan et al. 2024). As a result, the effectiveness of these dispersal barriers, which is likely to vary with time and taxa, remains untested. Further, several phylogenetic studies from Peninsular India may have conflated population divergence across barriers with speciation, though the data now available is insufficient to confirm or refute these assertions. Such assumptions may have resulted in taxonomic inflation and misleading conclusions regarding the roles of these physical barriers in promoting diversification.

In this context, we investigate the phylogeography of the Peninsular Indian flying‐lizard, *Draco dussumieri* Duméril & Bibron, 1837 (Figure 1b). The agamid genus *Draco* is unique among extant reptiles for its wing‐like patagium, which aids in generating lift during gliding. It is one of several vertebrate clades in Asia that likely evolved gliding to adapt to the tall Dipterocarp trees that form the predominant vegetation in much of the region's wet‐evergreen forests (Dudley and DeVries 1990; Heinicke et al. 2012; Chaitanya et al. 2023). Recent research has revealed strong correlations between the distributions and diversification patterns of *Draco* lizards and Dipterocarp trees (Chaitanya and Meiri 2022; Chaitanya et al. 2023). The distribution of *D. dussumieri* in Peninsular India encompasses the major biogeographic barriers discussed above but stops at the Goa gap (Figure 1a)—a vegetational barrier that demarcates regions with tall trees to the south and those bereft of them to the north (Chaitanya and Meiri 2022). We sampled from across the distribution range of *Draco dussumieri* (Figure 1a) to investigate phylogeography, potential speciation, and the roles of paleoclimate and established geographic dispersal barriers in shaping these patterns. We used mitochondrial DNA and genome‐wide SNPs to identify population clusters based on phylogeographic and clustering analyses. We then analysed hybridisation patterns and demographic history to establish lineage independence of candidate populations. We conducted explicit tests of isolation by distance (IBD) and isolation by environment (IBE) to understand how these processes influenced genetic variation. Further, we assessed potential multi‐locus adaptation to the environment using correlations between SNPs and environmental predictors as a preliminary test for adaptive differentiation. Finally, we used present‐day and historical climatic GIS data to ascertain the role of Pleistocene climatic fluctuations, estimate ecological niche divergence between identified genetic clusters, and to test if the spatial limit between candidate populations potentially represents a climatic barrier in the Western Ghats.

We considered two hypotheses that may explain diversification in *D. dussumieri* : (a) population structure is associated with geography—that is, phylogeographic breaks are expected to coincide with traditionally accepted geographic barriers and modelled ecological niches of these populations may or may not overlap significantly—(b) population structure is associated with ecological niche divergence—that is, phylogeographic breaks are expected to coincide with climatic transitions (rather than with geographic barriers), with limited overlap between ecological niches.

## 2 Materials and Methods

### 2.1 Field Sampling and Genetic Data

We conducted sampling across Peninsular India in two phases. To understand how genetic diversity within *D. dussumieri* is distributed in space, we first collected individuals from over 20 locations across known biogeographic barriers in the region. Next, we reconstructed a mitochondrial phylogeny (see Section 3) of these individuals to identify broad clades within *D. dussumieri* and their distributions. The mitochondrial clades informed our primary lineage hypothesis and further sampling strategy. In the next phase, we focussed our sampling effort at potential contact zones between candidate lineages to be able to understand the extent of admixture and gene flow between them. In total, we collected 60 individuals from 36 locations across the extant distribution of *D. dussumieri* in peninsular India (Figure 1a; Figure S1; File S1). Early identification of candidate lineages and their potential contact zones during our sampling helped prevent oversampling in eco‐sensitive regions like the Western and the Eastern Ghats. For instance, we refrained from sampling certain localities, especially in the Eastern Ghats (marked with an ‘X’ in Figure 1a), where populations were limited to only a few severely range‐restricted individuals. Sampling and fieldwork were conducted outside protected areas between the years 2014–2021 under permits (WL10‐4950/2014; WL5(A)/45296/2021; PCCF(WL)E2/CR‐10/2021‐22; D‐22(8)/WL/Research/CR‐108/2015‐16; MSBB/Desk‐5/Sect‐7/Research/NOC/CR‐299/389/16‐17; 4418/2014/WL‐3) issued by individual state forest departments.

Our mitochondrial dataset consisted of three gene fragments (NADH dehydrogenase subunit 2—ND2, 12S ribosomal RNA, 16S ribosomal RNA, totalling 1999 base pairs) for 54 individuals. Standard Sanger sequencing laboratory methods were used for extraction, PCR, and sequencing, and the methods used for processing and assembling these sequences are detailed in Methods S1.1. Primers and protocols used for sequencing are listed in Table S2.

We generated SNP data for 44 individuals from 32 localities across the distribution of *D. dussumieri* , ensuring several individuals from each of the identified mitochondrial clades were sequenced. These data were generated using Genotype‐By‐Sequencing (GBS) with the MseI restriction enzyme at Nucleome Informatics (www.nucleomeinfo.com). Libraries were sequenced on a Novaseq6000 high‐throughput sequencing platform (Illumina Inc.). Further details of the library preparation are provided in Methods S1.2. Raw sequences were examined for data quality issues using the program FastQC (Andrews 2010).

### 2.2 Phylogenetic Reconstruction Using Mitochondrial DNA

We acknowledge that inferring intraspecific relationships using a bifurcating tree is inappropriate, since reticulation resulting from gene flow between tip nodes cannot be represented. However, our intention was to identify broad clades within *D. dussumieri* that may provide a useful heuristic to understand how individuals from different locations cluster, and consequently help inform our primary lineage hypothesis.

We constructed an ultrametric tree of 54 individuals using the program BEAST2 (Bouckaert et al. 2014). The southeast Asian species *D. blanfordii* and *D. maculatus* were used as outgroups. We unlinked the site and clock models for the three mitochondrial genes to allow varying rates of substitution between them, but their tree models were linked to generate a single phylogeny that is representative of the lack of recombination and the linked nature of mitochondrial DNA. The best‐suited model of sequence evolution was estimated using the model‐test algorithm implemented in BEAST2. We used a relaxed, uncorrelated log‐normal clock model for each gene to allow for potential between‐lineage rate variation, and a Yule model of diversification (following Sarver et al. 2019) to reconstruct the phylogeny. The analysis was conducted using two runs of four chains (1 cold and 3 heated) each for up to 100 million generations, sampling every 10,000 generations from the posterior distribution of trees. We inferred convergence of the runs using Tracer v1.7.2 (Rambaut et al. 2018) when the effective sample sizes (ESS) for each parameter were > 200. The first 10% of the samples were discarded as burn‐in, and the tree with maximum clade credibility (MCC) was selected using the program TreeAnnotator v2.6.6 (Drummond and Rambaut 2007).

### 2.3 ddRAD Data Assembly

We performed bioinformatics of the sequenced ddRAD libraries using the program ipyrad v.0.9.95 (Eaton and Overcast 2020). First, raw reads with more than five bases with a phred Q‐score < 20 were excluded from further processing. Reads were clustered *de novo* within samples using the *vsearch* tool (ipyrad step 3) based on a clustering threshold that was optimised for our dataset (see below), and the resultant clusters were aligned using the MUSCLE algorithm (Edgar 2004). Clusters with a sequencing depth of less than six reads were discarded, as were consensus allele sequences estimated from clustered reads if they contained either more than 5% ambiguous sites (‘N's) or more than 5% heterozygous bases. The filtered sequences were clustered as homologous loci based on the chosen clustering threshold.

A methodological issue central to RAD‐sequenced data when a reference genome is unavailable is determining the optimal sequence similarity clustering thresholds to establish homologous loci for downstream analyses (Ilut et al. 2014; Harvey et al. 2015; McCartney‐Melstad et al. 2019). We constructed a set of six ddRAD assemblies by varying clustering thresholds from 85%–95% using loci shared between at least four individuals (min4). We then calculated four metrics suggested by McCartney‐Melstad et al. (2019) on each assembly to choose the optimal clustering threshold value for our dataset: (i) per‐individual percent heterozygosity, (ii) Pearson's correlation coefficient between pairwise genetic dissimilarity and data missingness, (iii) cumulative variance explained by the first eight principal components retained from a principal component analysis, and (iv) percentage increase in SNP divergence per 100 km (isolation by distance). We used R scripts available in McCartney‐Melstad et al. (2019) to calculate and plot these metrics. Per‐individual heterozygosity (i), cumulative variance explained by the first eight PCs (iii), and isolation by distance (iv) were maximised at a clustering threshold of 85%, while correlation between pairwise genetic dissimilarity and data missingness (ii) sharply increased at threshold values higher than 85% (Figure S2). Based on this consensus, we chose a clustering threshold of 85% for our assemblies to be used in further downstream analyses.

Stricter thresholds for missing data are not always optimal and tend to disproportionately exclude loci with the highest mutation rates (Huang and Knowles 2016). Consequently, assemblies with a low proportion of variable sites are likely to influence genetic clustering analyses and phylogeographic patterns. To test the effects of missing data, we constructed two assemblies with (i) 50% missing data, containing loci shared across at least 22 individuals (min22; 27,971 loci); and (ii) 25% missing data, containing loci shared across at least 33 individuals (min33; 2204 loci). While these assemblies differ in the number of loci and missingness, they both include all 44 individuals selected for genomic analyses. Details of these individual datasets are provided in Table S2.

### 2.4 Inferring Population Structure

To infer the number and composition of genetic clusters, and to account for the effects of missing data on these inferences, we used the assemblies with 50% (min22) and 25% (min33) missingness generated in the previous step.

We used a model‐based method (STRUCTURE) and two non‐model centric methods (*K*‐means clustering and PCA) to infer genetic clusters within *D. dussumieri* without a priori information on population structure. First, we used the program STRUCTURE v2.3.4 (Pritchard et al. 2000) to infer the number of genetic clusters and probabilistically assign individuals in our dataset to each identified cluster. STRUCTURE uses a Bayesian approach to infer population structure based on allele frequencies within and among populations in a dataset. We randomly sampled 6828 and 1406 unlinked SNPs from the min22 and min33 assemblies respectively, and conducted clustering analyses on these filtered datasets. We varied the number of clusters (K) from 1 to 5, where five was the maximum number of populations we expected based on our primary lineage hypothesis (see Section 3). We ran 10 replicate runs for each K for one million generations discarding the first 100,000 as burn‐in. We analysed both datasets under a correlated model of allele frequencies that allows for admixture between populations. However, since the correlated model tends to overestimate the optimal number of clusters (Falush et al. 2007), we reanalysed our data under an uncorrelated model to gauge how these models impact our inferences of population structure. Convergence of replicate runs were assessed based on the variance of the α parameter and log‐likelihood scores. Subsequently, we inferred the optimal number of genetic clusters based on three metrics: (i) Log‐probability of the data for a given *K*, that is, LnP(*K*) and its variance; (ii) the *∆K* statistic (Evanno et al. 2005) calculated using the second order derivative of LnP(*K*), implemented in STRUCTURE HARVESTER (Earl and VonHoldt 2012); and (iii) the unequivocal assignment of at least one sampled individual to each population cluster (i.e., inferred probabilistic ancestry ≈ 1.0) identified by the STRUCTURE algorithm (Pritchard et al. 2000). Results of the 10 replicate runs were summarised using CLUMPP v.1.1.2 (Jakobsson and Rosenberg 2007) and visualised using customised scripts written in R v4.1.2. To estimate population differentiation due to genetic structure, we calculated pairwise *F*_ST_ statistics for the clusters identified by STRUCTURE using the weighted Weir and Cockerham (Weir and Cockerham 1984) estimator implemented in VCFtools (Danecek et al. 2011). Individuals were assigned to populations based on the proportion of their ancestry, that is, Q > 0.5, as estimated by STRUCTURE.

In addition to STRUCTURE, we used the non‐model‐based algorithm *find.clusters* implemented in the R package *adegenet* (Jombart 2008) to infer the number of optimal genetic clusters in our datasets. This function transforms data using principal component analysis (PCA) and runs successive *K*‐means tests with an increasing number of clusters (K). For each K‐model, a goodness‐of‐fit test is computed, which allows us to choose the optimal number of clusters (*K*). We transformed the data using the first 40 principal component (PC) axes and sequentially increased the number of clusters (*K*) from 1 to 10. We chose the optimal *K* value for each dataset that minimised the Bayesian information criterion (BIC) score.

Finally, we conducted principal component analyses (PCA) on the assemblies using the program ipyrad.pca(). We allowed random subsampling of one SNP per locus to reduce the effect of linkage and subsampled loci that were present in at least 60% of the individuals. We ran 25 iterations of the PCA and plotted the averages of the first 2 PC axes.

Since all three clustering methods returned similar results for both ddRAD assemblies, we conducted subsequent analyses using the assembly containing the least missing data (min33).

### 2.5 Inferring Admixture Using Triangle Plots

Our STRUCTURE analyses found support for the presence of two geographically distinct populations (northern and southern) within *D. dussumieri* and a gradient of intermediate populations with mixed ancestry in the Central Western Ghats region (see Section 3). However, clustering methods such as STRUCTURE and PCA cannot differentiate admixture from continuous genetic variation that is characteristic of isolation by distance (Kong and Kubatko 2021; Wiens and Colella 2024a). As a preliminary test to distinguish between these processes and to assess the extent of potential hybridisation, we visualised covariance of hybrid index (*S*: proportion of alleles descending from one parental lineage) with interclass heterozygosity (*H*: proportion of an individual's loci that have one allele from each ancestral lineage) for each individual using the R package *triangulaR* (Wiens and Colella 2024b). The package gleans ancestry‐informative markers using SNP datasets, jointly calculates *S* and *H* for each individual that are then visualised in a triangle plot in the context of theoretical expectations under Hardy–Weinberg equilibrium (HWE). Triangle plots allow accurate detection of recent admixture when populations follow expectations of HWE. However, when assumptions of HWE are violated, triangle plots cannot readily distinguish admixture (recent or late‐generation) from a history of isolation by distance (see Wiens and Colella 2024a). Admixed individuals (*S* ≈ 0.5) with *H* = 1 indicate first filial (F1s) and *H* = 0.5 s filial (F2s) or later hybrids. When assumptions of HWE are violated, that is, under scenarios of selection, drift or inbreeding which result in a loss of allelic variation, individuals with mixed ancestry are plotted below the HWE curve (*H* < 0.5). We used an allele frequency difference threshold of 0.75 on the min33 dataset (2204 SNPs) resulting in a down sampled dataset of 643 ancestry‐informative markers for this analysis. Individuals were assigned to populations based on their ancestry proportions (Q) following the results of STRUCTURE and colour‐coded for visualisation.

Further, we visualised the spatial distribution of individuals with mixed ancestry by plotting interclass heterozygosity (*H*) against latitude.

### 2.6 Genetic Variation and Its Relationships With Distance and the Environment

To explicitly test if IBD explains genetic variation seen within *D. dussumieri* , we first conducted a mantel test implemented in the package *dartR* (Gruber et al. 2018). We filtered unlinked SNPs by removing sites that were non‐biallelic or had a minor allele frequency < 0.05. We used linearised *F*_ST_ (*F*_ST_ = *F*_ST_/1 − *F*_ST_) and Euclidean distance of spatial coordinates as measures of genetic and geographic distances respectively. We permuted the analysis 999 (default) times.

To compare the effects of IBD and IBE on genetic variation, we used generalised dissimilarity modelling (GDM) in the R package *gdm* (Ferrier et al. 2007; Mokany et al. 2022). We used the pairwise *F*_ST_ matrix generated by *dartR* as our measure of genetic distance (response variable) and 11 continuous environmental variables (downloaded from CHELSA, Karger et al. 2017, 2021), elevation, and pairwise geographic distances as predictors (see Table 1; Data S1). We fitted a GDM to these data and assessed variable inclusion in the model (spline coefficients > 0). We assessed model fit (percent deviance explained), importance, and significance of each variable in predicting genetic variation, using 1000 permutations.

Finally, to test if divergent selection may be at play between lineages because of potential adaptation to local environments, we conducted redundancy analysis (Forester et al. 2018; Capblancq and Forester 2021) and evaluated preliminary genotype–environment associations (GEA). This uses constrained ordination that assesses the explanatory power of multivariate predictors acting on multivariate responses such as genotypes. We used unlinked, genome‐wide SNPs and six weakly intercorrelated bioclimatic variables from the dataset used in the IBE analysis to identify loci that are potentially adaptively differentiated as a response to each environmental predictor. We further evaluated the number of candidate loci potentially correlated with each predictor and the strengths of such correlations. However, our dataset is limited compared to the standards necessary for genome‐wide association studies (Murcray et al. 2011). Further, due to the lack of a reference genome, we cannot determine the physical location of these loci and thus cannot make comparative assertions on adaptive potential. Our GEA analysis is therefore preliminary and only aims to identify any variations, if present, that are associated with adaptive ecological differentiation.

### 2.7 Demographic History and Gene Flow

We used the program GADMA (Noskova et al. 2020, 2023) to reconstruct demographic models by estimating divergence time, population growth, and the timing and magnitude of gene flow between identified population clusters. We used the “moments” engine (Jouganous et al. 2017) which implements a genetic algorithm for the global search of parameter space and the unsupervised inference of demographic history from allele frequency spectrum (AFS) data. Individuals were assigned to either the northern or southern populations based on the proportion of their ancestry, that is, Q > 0.5, as estimated by STRUCTURE. We assigned two time‐intervals post divergence (initial: [1,1], final: [1,2]) to infer potential changes in the demographic histories of lineages. To calculate absolute divergence time, GADMA requires the specification of mutation rates beforehand. This is a contentious issue, especially for studies at a phylogeographic scale where closely related fossils for calibration are unavailable (Schenk 2016). We opted to use the widely accepted nuclear DNA mutation rate of 1 × 10^−9^ substitutions/site/year in accordance with many recent squamate studies (Brandley et al. 2011; Allio et al. 2017; Blom et al. 2017) including studies on other species of *Draco* (Reilly et al. 2022; McGuire et al. 2023). Other studies on reptile diversification have also used several faster mutation rates ranging from 2.2 × 10^−9^ to 1 × 10^−8^ substitutions/site/year (Gehara et al. 2017; Harrington et al. 2018). We acknowledge the uncertainty associated with using a single, overarching mutation rate. However, these faster rates, if estimated to be closer to germline mutation rates, would only result in younger estimated divergence times and are therefore unlikely to affect the broader inferences we make in this study. To account for sensitivity of the model to mutation rates, we reconstructed demographics using the fastest mutation rate available from literature for squamates (1.0 × 10^−8^ substitutions/site/year). Further, to account for sensitivity of our results to model structure, we repeated the analysis allowing for four time‐intervals post divergence (i.e., initial: [1,1], final: [1,4]). We assumed a generation time of one year for *D. dussumieri* based on natural history data from the Southeast Asian congener *Draco spilopterus* (Alcala 1967), and used an effective sequence length of 614,152 basepairs. We launched 10 repetitions of the parameter optimisation algorithm for each run and selected models with the highest log‐likelihood values and AIC scores.

### 2.8 Climatic Variables and Occurrence Data

We used historical and present‐day climate data to analyse climatic niche divergence/overlap between the northern and southern lineages inferred from our genetic clustering analyses, and to test the possible effects of Pleistocene climatic fluctuations on their distributions. We downloaded raster datasets of 15 bioclimatic variables at a spatial resolution of 30 arc sec (~1 km) for the recent past (averaged between the years 1979–2013) and the NCAR‐CCSM4 general circulation model for the Last Glacial Maximum (from ca. 21 ka) from CHELSA (Karger et al. 2017, 2021). We used the LGM model because there are no fine‐scaled paleoclimatic data for earlier glacial maxima (Karger et al. 2017, 2021). We therefore treated this dataset as a general indicator of conditions that persisted during Pleistocene glacial maxima rather than as a precise reconstruction of the actual conditions during population divergence or other demographically significant events.

Variables linking precipitation and temperature such as mean temperatures of the wettest and driest quarters (BIO8 and BIO9) and mean precipitation of the warmest and coldest quarters (BIO18 and BIO19) were not considered for analyses as they potentially leave spatial artefacts in the data (Campbell et al. 2015). The remaining variables were tested for collinearity using the Pearson's correlation coefficient (|*r*| > 0.80) between variable pairs to identify strongly correlated variables. Our final dataset for climatic modelling retained seven weakly intercorrelated variables that were relevant to the questions we ask. To compare present‐day with historical climatic suitability for *D. dussumieri* , we used the variables that represent annual means and variances (seasonality) in temperature and precipitation (means—BIO1 and BIO12 and seasonality—BIO4 and BIO15, respectively). Since *Draco dussumieri* are strictly diurnal, we retained mean diurnal range and isothermality (BIO2 & BIO3 respectively). We included maximum temperature of the warmest month (BIO5) to account for extreme, potentially limiting temperature change for a wet‐adapted tropical lizard during the LGM. All raster layers were clipped to accommodate the extant distribution of *Draco dussumieri* in Peninsular India (8° N–16° N; 72° E–81° E).

We obtained 172 occurrence records for *Draco dussumieri* from the Global Assessment of Reptile Distributions (GARD) database (Roll et al. 2017; Caetano et al. 2022) supplemented by our observations from the field. Multiple records within a 1 km^2^ radius of each other were trimmed to ensure that each predictor raster cell contained no more than one presence record. We attributed an occurrence record to a population as inferred by STRUCTURE, only if the region was unambiguously assignable to its inferred distribution. The final datasets contained 41 and 65 occurrence records for the northern and southern lineages, respectively. We verified that occurrence points for each lineage were above the minimum number required for constructing robust niche models for our geographical extent and the predictor layers used for modelling (van Proosdij et al. 2016).

### 2.9 Climatic Niches, Niche Divergence, and Range‐Breaks

We constructed maximum entropy climatic niche models for the northern and southern lineages using MaxEnt version 3.4.3 (Phillips et al. 2006). To test for niche divergence between populations and the impacts of LGM climate change on them, occurrence data for these lineages were used separately to model present distributions and projected onto the LGM dataset. Model performance was assessed using area under the receiver operating characteristic curve (AUC) and the true skill statistic (TSS) metrics. Parameters used for MaxEnt modelling are detailed in the Methods S3.1.

To assess niche divergence, we used the Schoener's *D* metric (Schoener 1968) on the estimated MaxEnt suitability maps for the present and the LGM, estimated using ENMTools 1.0 (Warren et al. 2021). The metric ranges from 0 to 1, where 0 indicates no niche overlap and 1 indicates perfectly identical niches between the two groups.

To test whether the geographic boundary between *D. dussumieri* populations coincides with a sharp environmental transition zone, (i.e., the boundary represents a climate‐driven biogeographic barrier), we conducted range‐break tests implemented in ENMTools using the present day and the LGM climatic data. In this test, the empirically estimated overlap (Schoener's *D*) between niches of two species is compared to a null distribution of niche divergence, which is constructed by randomly varying the location and shape of the geographic boundary that separates them. Rejection of this null supports the alternate hypothesis that the empirical boundary between the species' distributions occurs at an environmental transition zone and thus represents a biogeographic barrier. We created a null distribution of the Schoener's *D* metric between the northern and southern lineages using 100 random replicates using the ‘blob’ range‐break method available in ENMTools (Glor and Warren 2011). We then compared the empirically calculated Schoener's *D* with the null distribution and rejected the null hypothesis if the empirical value was either outside the lower end of the distribution, or smaller than 95% of the values (i.e., *p* < 0.05) in the distribution.

## 3 Results

### 3.1 Phylogeographic Inference Using Mitochondrial Data

**FIGURE 2:**
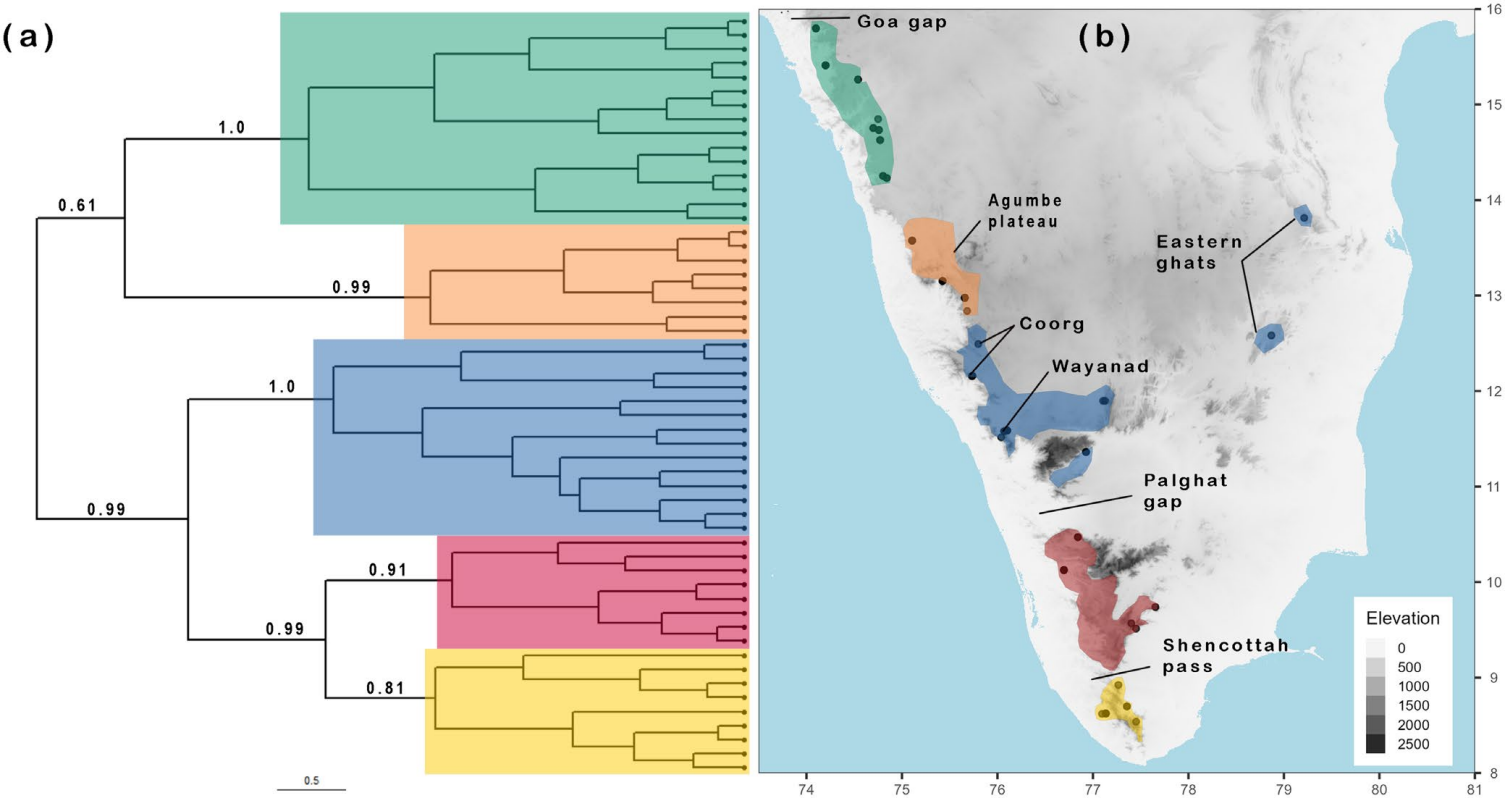
Mitochondrial phylogeny (a) of *D. dussumieri* (outgroup removed) with branch support values for the clades that inform our primary lineage hypothesis. Sampling locations for mitochondrial DNA data (b) colour coded according to clades. Distribution ranges of clades are either informed by our understanding of biogeographic barriers or follow sampling locations. Gaps between clades represent regions where *D. dussumieri* has not been collected or is presumed not to occur. The phylogenetic pattern in *D. dussumieri* follows a latitudinal gradient across Peninsular India.

The reconstructed mitochondrial ultrametric tree revealed a latitudinal phylogenetic pattern (Figure 2a). We demarcated five well‐supported (posterior probabilities, PP: 0.81–1.0) clades which we inferred to be potential candidate lineages based on recognised biogeographic barriers in peninsular India. The pattern shows cladogeneses across the Shencottah pass and the Palghat gap. However, individuals from the Eastern Ghats clustered strongly (PP:1.0) with the Western Ghats clade distributed immediately north of the Palghat Gap (Figure 2a, in blue). We demarcated a strongly supported (PP: 0.99) Central Western Ghats clade containing individuals from the Agumbe plateau, and a northern clade, which extends up to the Goa gap (15.8*°* N).

### 3.2 Population Structure in *D. dussumieri*

**FIGURE 3:**
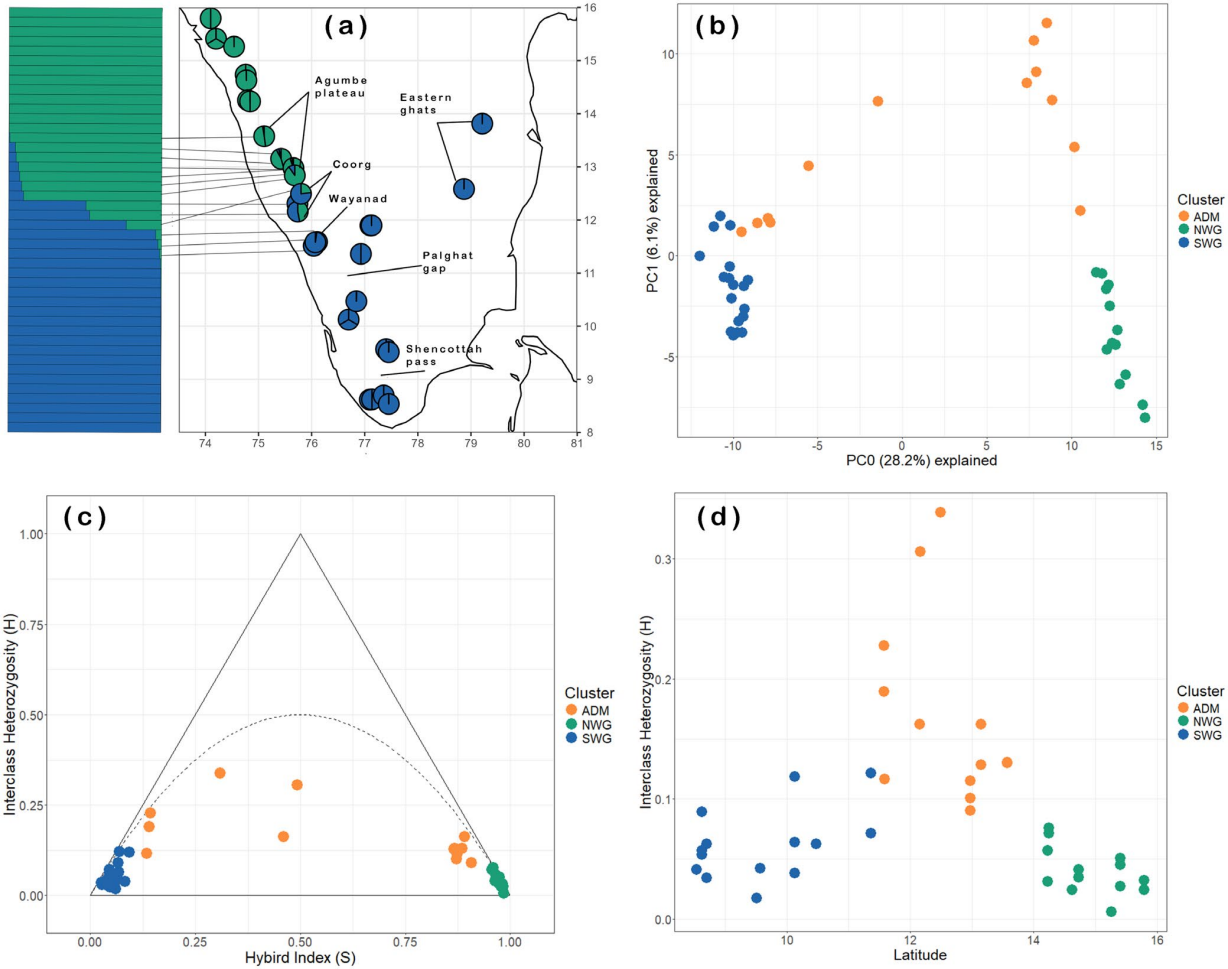
Results of the STRUCTURE analysis (a) using the dataset with loci shared across at least 33 individuals (min33), mapped against sampling locations. The admixed individuals occur in the Wayanad, Coorg and Agumbe plateau regions (between 11.5*°* N–13.5*°* N) in the Western Ghats. The first two principal components (b) averaged from 25 replicate PCAs conducted on the min33 dataset, showing genetic segregation of the northern, southern,and admixed lineages. Triangle plot (c) using SNP data that maps hybrid index (S) against interclass heterozygosity (H). Individuals plotted on the curve follow theoretical expectations of Hardy–Weinberg equilibrium. Interclass heterozygosity of individuals plotted against latitude (d) in the Western Ghats shows that the more heterozygous individuals occur between 11.5*°* N–13.5*°* N. ADM, admixed individuals; NWG, Northern Western Ghats lineage; SWG, Southern Western Ghats lineage.

STRUCTURE analyses conducted on the min22 (50% missingness) and min33 (25% missingness) datasets returned results consistent with each other. All three methods of model evaluation identified a two‐population model (*K* = 2) as the best fit to both datasets. Log probability (LnP(*K*)) values for *K* = 2 and *K* = 3 were largely similar between runs (except for the correlated allele frequency model under the min22 dataset, where *K* = 2 had much higher values). However, ∆*K* values for *K* = 2 were significantly higher under both assemblies (Figures S3 and S4). Additionally, all models where *K* > 2 failed to unequivocally assign (ancestry proportion Q ≈ 1.0) at least one individual to each identified cluster (Figure 3a; Figure S5). The STRUCTURE analyses identified two, geographically cohesive, southern and northern population clusters, and intermediate populations showing clinal mixed ancestry in the Central Western Ghats region between Wayanad and the northern edge of the Agumbe plateau (ca. 11.5*°* N–13.5*°* N; Figure 3a). Ancestry proportions (Q) in the Central Western Ghats followed a latitudinal pattern where individuals from the southern parts of the Central Western Ghats such as Wayanad retained a significant proportion of their ancestry from the southern cluster, and vice versa. The Coorg plateau, which is situated roughly at the cline centre, contained individuals with more similar proportions of ancestry from the peripheral northern and southern populations. The southern cluster showed no population structure across established biogeographic barriers in Peninsular India, that is, the Palghat and Shencottah valleys and across the drier plains separating the Western and Eastern Ghats.

The *K*‐means tests conducted using *find.clusters*() identified *K* = 2 as the best fit to the min22 dataset based on BIC scores (Figure S6). But for the min33 dataset, the analysis returned marginally higher BIC scores for the *K* = 2 model, which nevertheless was insufficient to select the K = 3 model as optimal. The PCA on the min22 (Figure S7) and min33 datasets (Figure 3b) returned patterns of clustering that were indicative of either admixture or isolation by distance (see Wiens and Colella 2024b). The peripheral northern and southern populations, as identified by STRUCTURE, were clearly differentiated along PC0, which explained most of the genetic variation (28%). Individuals with mixed ancestry were plotted closer to peripheral populations that made up a high proportion of their ancestry along PC0, while individuals from the cline centre (Coorg plateau) with more similar ancestry proportions from peripheral populations occupied relatively more central positions. Finally, weighted pairwise *F*_ST_ estimates between southern and northern populations revealed significant differentiation (*F*_ST_ = 0.27) under both the min22 and min33 assemblies.

### 3.3 Inferring Admixture Using Triangle Plots

The southern and northern populations identified by STRUCTURE were assigned *S* values close to 0 and 1 respectively, and the lowest *H* values (Figure 3c). Individuals from intermediate populations with mixed ancestry were plotted closer to peripheral populations that made up a significant proportion of their ancestry. Individuals from the cline centre had higher interclass heterozygosities (*H*: 0.16–0.34) and hybrid indices closer to 0.5 (*S*: 0.3–0.49). All individuals with mixed ancestry were plotted below the curve (*H* < 0.5) which is consistent with deviations from HWE. This pattern suggests either recent or late‐generation hybridisation and non‐equilibrium conditions such as inbreeding, selection, or genetic drift resulting in a loss of allelic variation (Wiens and Colella 2024b). However, a history of isolation by distance cannot be ruled out, since triangle plots are unable to distinguish isolation by distance maintained over several thousand generations from a scenario of admixture where HWE assumptions are violated, as demonstrated using forward‐time simulations of these processes (see Wiens and Colella 2024b).

Individuals with the most interclass heterozygosity occurred in the Coorg Plateau (ca. 12*°* N; Figure 3d) which lies at the cline centre of individuals with mixed ancestry. Heterozygosities diminished in individuals further away from the Coorg plateau as ancestry coefficients tended proportionally more towards northern or southern populations.

### 3.4 Isolation by Distance, Isolation by Environment, and Adaptive Potential

**FIGURE 4:**
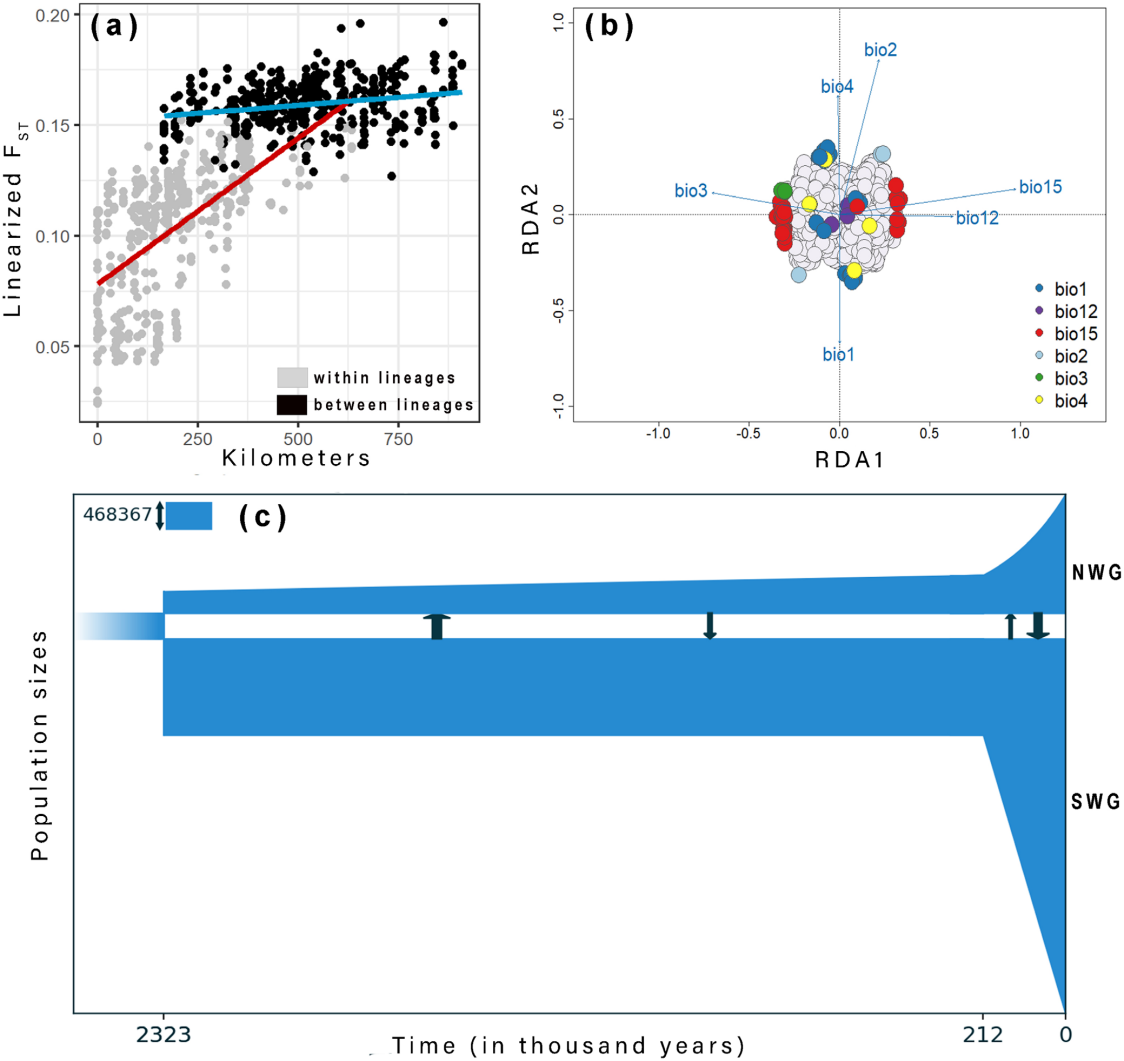
Pairwise *F*_ST_ as a function of geographic distances (a) between all individuals from within (grey) and between (black) lineages. Trend lines fitted using the R *lm* function show a discontinuous relationship among comparisons between and within lineages, a pattern suggestive of restricted gene‐flow supporting the presence of two species (see Prates et al. 2024). Genotype‐environment associations (b) that show SNPs that may be adaptively differentiated (significant correlations are colour coded) along axes of climatic predictors. Precipitation seasonality (bio15) was correlated with the most SNPs (34). The best‐fit population demographic model from GADMA (c) constructed using a mutation rate of 1.0 × 10^−9^ substitutions/site/year and two time‐intervals post initial divergence, showing population sizes (inset scale), times of key demographic events (divergence, population expansions, and migrations), and the strengths and directions of migration events (represented by black arrows). NWG, Northern Western Ghats lineage; SWG, Southern Western Ghats lineage.

Analyses of IBD among individuals showed a general positive correlation between genetic (linearised *F*_ST_) and geographic distances (*R* ^2^ = 0.523; *p* = 0.001). However, pairwise comparisons within and between lineages formed discontinuous trends of IBD and were reflective of a pattern of restricted gene flow between lineages (Figure 4a; see Prates et al. 2024).

The generalised dissimilarity model comparing the effects of distance and the environment on genetic variation was a good fit to the data (percent deviance explained: 80.7; *p* < 0.01). The model dropped bio1, bio6 and bio10 based on zero sum of coefficients and only estimated geographic distance and precipitation seasonality (bio15) as significant predictors (Table 1). Precipitation seasonality (bio15) was six times more important than geographic distance in explaining genetic distances between individuals, suggesting stronger support for isolation by environment rather than isolation by distance.

**TABLE 1:** Variable importance and their significance as estimated by the generalised dissimilarity model (GDM).

Predictors	Importance	*p*
**Precipitation seasonality (bio15)**	**6.133**	**0.02**
**Geographic distance**	**1.083**	**< 0.01**
Evapotranspiration	0.933	0.37
Mean temperature of coldest quarter (bio11)	0.827	0.44
Max temperature of warmest month (bio5)	0.602	0.55
Mean diurnal range (bio2)	0.401	0.84
Elevation	0.373	0.82
Mean annual precipitation (bio12)	0.352	0.68
Temperature seasonality (bio4)	0.331	0.72
Isothermality (bio3)	0.221	0.70

*Note:* Variables identified as significant are highlighted.

Clustered ordination using redundancy analysis identified 83 unique loci that were significantly correlated with the six environmental predictors (Figure 4b). Among these, precipitation seasonality (bio15) explained potentially adaptive variation in the most loci (34), followed by mean annual temperatures (bio1: 29 loci) and temperature seasonality (bio4: 11 loci). The other variables (bio2, bio3 and bio12) were correlated with three different loci each.

### 3.5 Demographic Modelling and Gene Flow

The GADMA analyses using a mutation rate of 1.0 × 10^−9^ substitutions/site/year and allowing for both two and four time‐intervals after the initial population split (Figure 4c; Figure S8) converged on the same preferred model with two time‐intervals and similar demographic scenarios of population expansions and migrations. This best model suggested early Pleistocene (2.3–2.2 Ma) divergence between the northern and southern lineages. Effective population sizes for both lineages were initially relatively small but grew significantly (3×–5×) during the mid‐Pleistocene (ca. 220–210 ka). Migrations were continuous between lineages after divergence with relatively lower initial rates (2 Nm: S → N 0.47–0.48; N → S 0.08) that increased significantly after populations expanded during the mid‐Pleistocene (2 Nm: S → N 0.58–0.74; N → S 0.47–0.48). Migration rates were nonetheless consistently below thresholds required to homogenise lineages (2 Nm = 1). The analysis using faster mutation rates (1.0 × 10^−8^ substitutions/site/year) resulted in similar population sizes and growth, migration rates and predictably estimated ca. 10 times younger dates for initial divergence and population expansion (Figure S8). We rely on the results of the analysis using a mutation rate of 1.0 × 10^−9^ substitutions/site/year (the most used rate in literature on squamate diversification) to infer absolute times of divergence and population expansions.

### 3.6 Climatic Niche Divergence and Range‐Breaks

**FIGURE 5:**
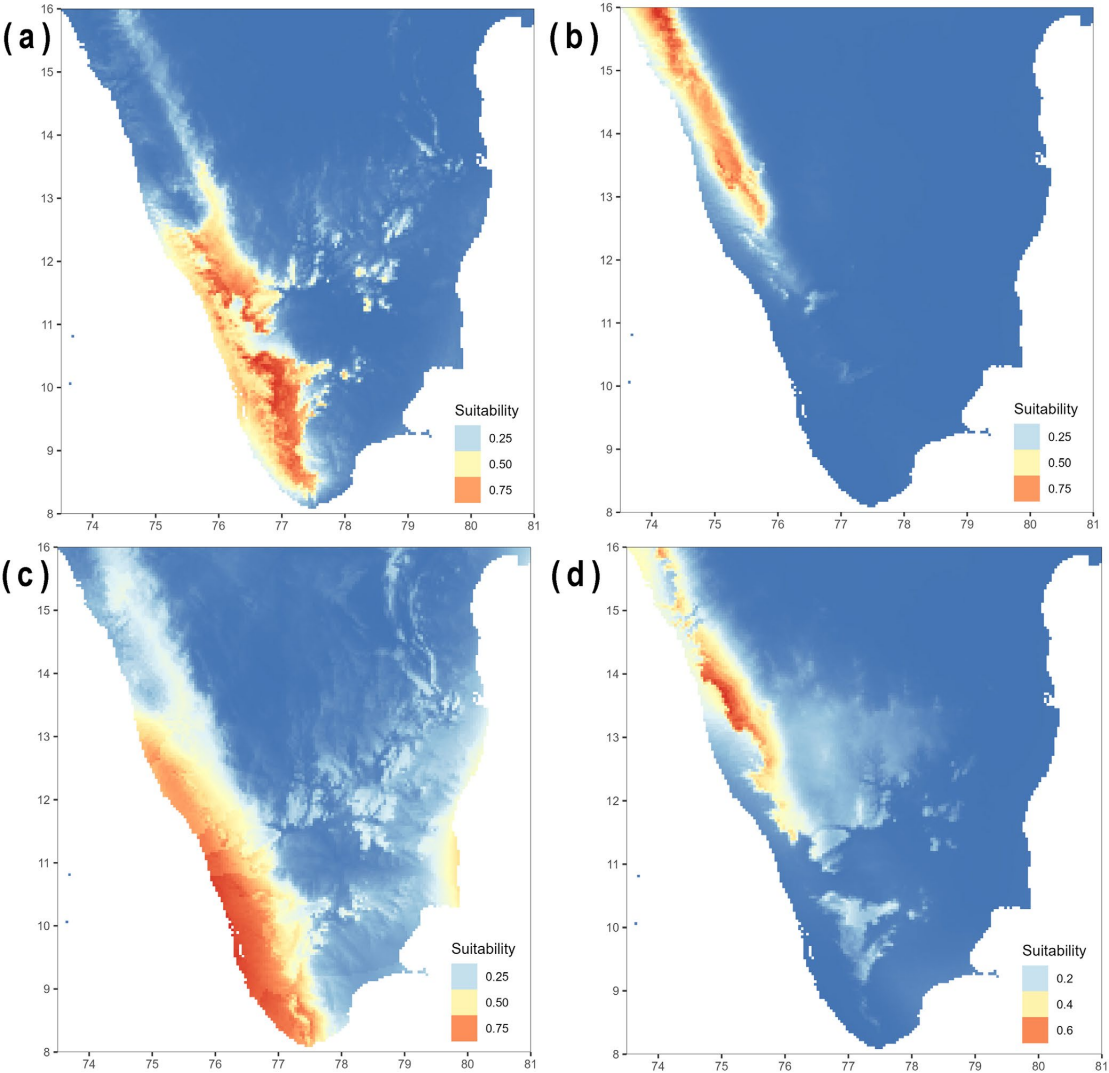
Climatic niche models of the southern (a, c) and northern (b, d) lineages constructed using six weakly correlated climatic variables during the present (top panel) and the Last Glacial Maximum (LGM; bottom panel). Suitable ranges were greater for both lineages during the LGM when compared to the present.

MaxEnt suitability models constructed for the northern and southern lineages returned accurate predictions under both criteria used for model evaluation (Table S3). They indicate that climatic niche divergence for the northern and southern lineages is significant (Schoener's *D*: 0.17) during the present and was moderate during the LGM (Schoener's *D*: 0.40). During the LGM, mean suitability in Peninsular India increased significantly for the southern (ca. 63%; Figure 5a,c) and moderately for the northern (ca. 18%; Figure 5b,d) lineages.

**FIGURE 6:**
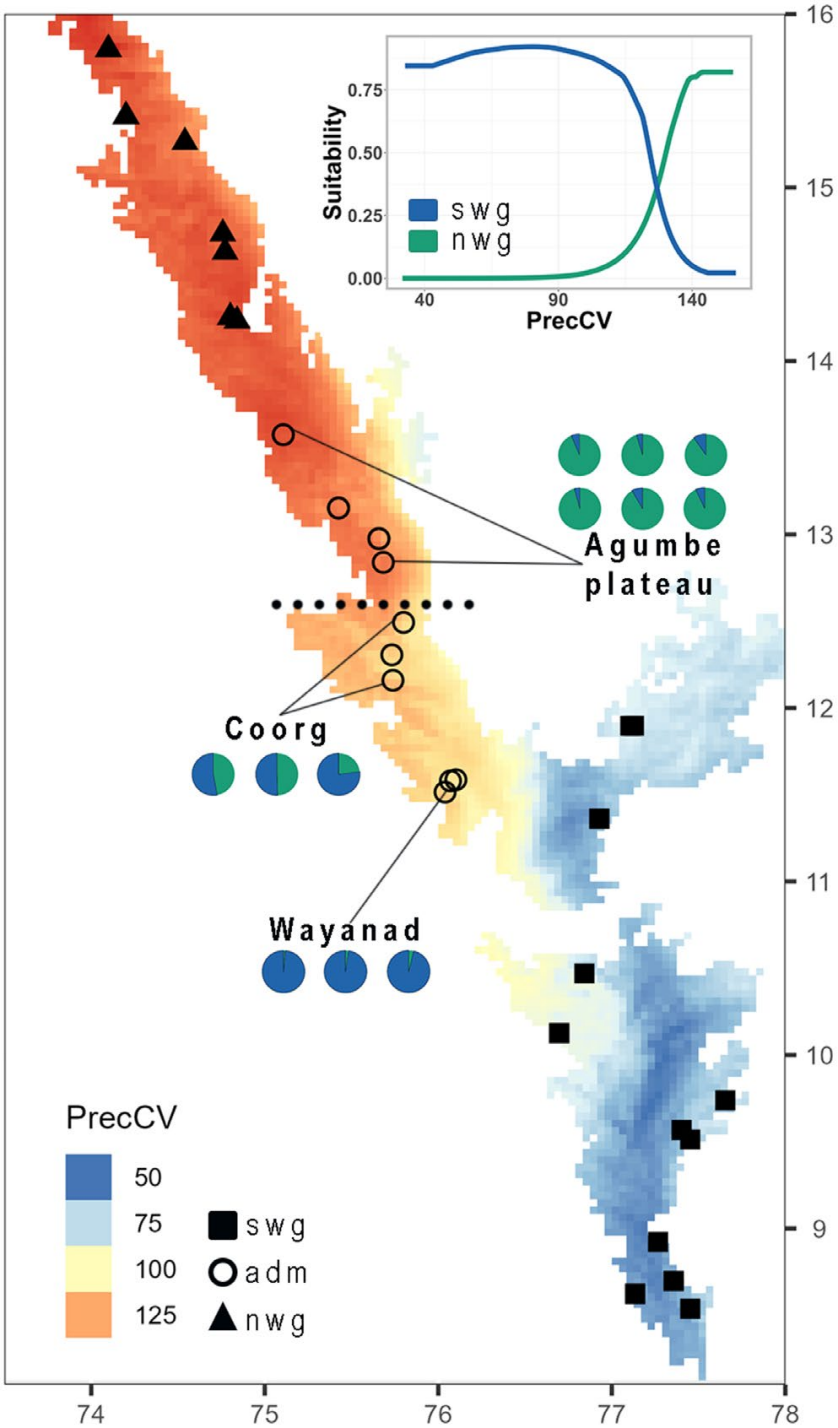
Distributions of the northern and southern lineages of *D. dussumieri* (sensu lato) in context of precipitation seasonality (coefficient of variation) in the Western Ghats. Pie charts show ancestry proportions of admixed individuals in the Central Western Ghats. The range break between northern and southern lineages is indicated by a dotted line, which further separates regions with high versus moderate‐to‐low rainfall seasonality. The inset shows MaxEnt mean response curves (averaged from 30 replicates) to precipitation seasonality for both lineages. ADM, admixed populations; NWG, Northern Western Ghats lineage; SWG, Southern Western Ghats lineage.

Precipitation seasonality (bio15) and mean annual precipitation (bio12) contributed the most to the suitability model for the northern lineage (Table 2). Precipitation seasonality emerged as the most significant variable when used in isolation and when omitted (File S2). Climatic seasonality (bio4 and bio15) variables contributed over 44% to the model constructed for the southern lineage, and the response curves suggest a preference for stabler climates (File S3). Temperature seasonality (bio4) contributed the highest gain when used in isolation, and precipitation seasonality (bio15) caused the most decrease in gain when omitted. Responses to precipitation seasonality were highly dissimilar (but not to temperature seasonality; Figure 6 inset) between northern and southern lineages.

**TABLE 2:** Mean percentage contributions of climatic variables (averaged over 30 replicates) to the MaxEnt models constructed for the northern and southern lineages of *D. dussumieri* .

Variable	Northern	Southern
Precipitation seasonality (bio15)	**78.6**	**13.6**
Mean annual precipitation (bio12)	**12.4**	**28.5**
Mean annual temperature (bio1)	5.2	5.4
Mean diurnal range (bio2)	1.7	6.3
Max temperature of the warmest month (bio5)	0.7	**13.7**
Temperature seasonality (bio4)	1.3	**31.2**
Isothermality (bio3)	0.1	1.2

*Note:* Variables with significant contributions are in bold.

The range‐break test supported the hypothesis (*p* = 0.04) that the present‐day boundary between the northern and southern lineages represents a biogeographic barrier which occurs at a climatic transition zone in the Western Ghats (Figure 6). However, this barrier was not significant (*p* = 0.68) during the LGM, and likely facilitated further dispersal and admixture of the two lineages.

## 4 Discussion

Our results strongly support the presence of two geographically cohesive lineages within *Draco dussumieri* (sensu lato) that are distributed in the north and the south (including populations in the Eastern Ghats) of the Western Ghats, respectively (Figure 3a–d). We further detected a gradient of admixed populations in the Central Western Ghats that roughly span 11.5*°* N–13.5*°* N latitudes. The best demographic model (Figure 4c) suggests an early Pleistocene divergence (ca. 2.3 ma) followed by continuous migrations between lineages, that increased in magnitude after populations expanded in the mid–late Pleistocene (ca. 200 ka). The between‐lineage *F*_ST_ estimate (*F*_ST_ = 0.27) exceeded the divergence threshold indicative of conspecificity (*F*_ST_ < 0.19) in a wide range of organisms (Roux et al. 2016). Migration rates between lineages inferred using GADMA (Figure 4a) were consistently less than one migrant per generation (2 Nm < 1) throughout their histories, suggesting the lack of homogenising gene flow after initial divergence (Burbrink et al. 2021; Dufresnes et al. 2021). Further, the IBD test revealed a discontinuous relationship between pairwise *F*_ST_ and geographic distance among individuals between and within lineages (Figure 4b), indicative of reproductive isolation with restricted gene flow (see Prates et al. 2024). Based on these estimates and drawing from population genetic theory, we establish lineage independence of the northern and southern populations, and propose that they represent distinct species.

Analyses of environmental niches of the northern and southern lineages show greater overlap during the LGM (Schoener's *D*: 0.40) than at present (Schoener's *D*: 0.17). They further suggest larger range sizes for both lineages during the LGM than at present (Figure 5). The range‐break tests find support for the hypothesis that the boundary separating the northern and southern lineages represents a climatic barrier in present‐day conditions (*p* = 0.04), but not during the LGM (*p* = 0.68). These results allude to greater climatic homogeneity in the Western Ghats during glacial maxima that facilitated expansions in populations and range sizes, which in turn may have resulted in increased migration rates between northern and southern populations. This ties in broadly with our demographic model, which suggests that population expansions and elevated migration rates roughly coincide with the penultimate glacial period (ca. 200–140 ka; corresponding to Marine Isotope Stage 6), which was likely one of the strongest and coolest among Quaternary glaciations (Jouzel et al. 1993; Colleoni et al. 2016; Hughes and Gibbard 2018). However, our data may be inadequate to infer more fine‐scaled patterns of population expansions and contractions in the context of changing climates.

Based on these findings, we infer that ancestral *D. dussumieri* likely underwent climate‐induced parapatric speciation, forming lineages in the north and the south of the Western Ghats during the early Pleistocene (ca. 2.3 Ma). These lineages may have adapted in situ to local environments, especially to highly dissimilar rainfall regimes (Figure 6). Migrations between lineages post‐divergence were continuous and migration rates increased after each population increased in size and expanded its range (Figure 4c). This was likely facilitated by more homogeneous climate across the Western Ghats during glacial cycles. Gene flow during migrations eroded divergence, but was nevertheless insufficient to homogenise lineages. We postulate that alternating climatic cycles during the Pleistocene regulated population sizes, range sizes and the magnitude of gene flow between lineages across time, resulting in the phylogeographic pattern in *D. dussumieri* (sensu lato) that we see today.

This is the first study from the Western Ghats biodiversity hotspot to use genome‐wide data and contact zone analyses to unravel phylogeographic patterns and the processes that caused them, in a widely distributed, wet‐adapted organism. It illustrates the challenges in discerning “structure versus species” in closely related, morphologically cryptic, sister lineages from an ecologically complex landscape such as the Western Ghats.

### 4.1 Potential Adaptation to Local Rainfall Regimes

Precipitation seasonality (bio15) emerged as the most important predictor (roughly six times higher than geographic distance) of genetic variation across individuals in the generalised dissimilarity models (Table 1). No other predictor was identified as significant. Additionally, genotype‐environment associations estimated by our redundancy analysis reveal that most of the loci that may be adaptively differentiated are correlated with precipitation seasonality, followed closely by mean annual temperatures (Figure 4c). Climatic niche models for both lineages detect precipitation seasonality and mean annual precipitation as among the most significant predictors contributing to model gains (Table 2; Files S2 and S3). Response curves indicate that higher rainfall seasonality is more suited (predicted probability of presence: 0.80) for the northern lineage, while the southern lineage prefers regions with stabler rainfall patterns (Figure 6 inset).

Further, the range‐break between the northern and southern lineages lies between regions with high and moderate‐to‐low precipitation seasonality, which generally increases with latitude in the Western Ghats (Figure 6). The Agumbe plateau, which marks the southern boundary of the northern lineage, is one of the wettest regions in the world, receiving > 7000 mm of annual rainfall, over 90% of which is received during just three summer months (Revadekar et al. 2018). On the other hand, the Southern Western Ghats is relatively less seasonal, receiving lesser rainfall (3000–5000 mm) that is distributed across 7 months (Revadekar et al. 2018). The Coorg plateau in the Central Western Ghats where the range‐break occurs, is a transition zone between regions with high and low precipitation seasonality (Figure 6).

Among all the factors we identified, precipitation seasonality is the correlate that most likely explains genetic variation and ecological isolation between the northern and southern lineages, and may have influenced adaptive differentiation to local rainfall regimes. Further, we hypothesise that the rainfall transition zone in the Central Western Ghats region (i.e., where the range‐break between *Draco* lineages occurs) may serve as a ‘suture zone’ for several widespread, wet‐adapted species in the Western Ghats, where phylogeographic breaks within them will spatially coincide.

### 4.2 Phylogeographic Patterns in Context of the Peninsular Indian Landscape

The generally acknowledged biogeographic blueprint in Peninsular India involves clade endemism of wet‐adapted taxa that coincide with geographic barriers to dispersal, that is, the Palghat and Shencottah valleys, and less frequently, with the arid plains between the Western and the Eastern Ghats (but see Ramachandran et al. 2017; Chaitanya and Meiri 2022). Several studies implicate these valleys as major biogeographic barriers for birds (Robin et al. 2010), large mammals (Vidya et al. 2005; Kolipakam et al. 2019), rodents (Varudkar and Ramakrishnan 2015), primates (Ram et al. 2015), frogs (Van Bocxlaer et al. 2012), geckos (Chaitanya et al. 2019; Agarwal et al. 2019) and snakes (Mallik et al. 2020, 2021). Our mitochondrial phylogeny recovers clades consistent with these biogeographic breaks (Figure 2a). However, our genetic clustering tests using genome‐wide nuclear SNPs reveal little structure within the southern lineage that spans all these prominent barriers (Figure 3a), suggesting that they may have been permeable at different periods in deep time, allowing for homogenising gene flow between populations on either side. Such discordances could occur simply because mitochondrial DNA is more readily fixed in populations than nuclear DNA is (Brown et al. 1979; Ballard and Rand 2005; Toews and Brelsford 2012). This disparity is due to several reasons, including the four times lower effective population sizes (N_e_) for mitochondrial DNA, resulting in fixation times that are at least fourfold faster than that of the nuclear genome (Brown et al. 1979; Moore 1997). Therefore, biogeographic inferences made strictly using (or under the strong influence of) mitochondrial divergence as evidence for population structure and/or speciation must be interpreted with caution, since even recent homogenising gene flow between populations may be obscured by faster mitochondrial fixation rates.

Moreover, simplifying the biogeography of the Western Ghats to associate phylogenetic breaks in taxa with physical barriers does not capture the remarkable ecological complexity of this landscape. For instance, Chaitanya and Meiri (2022) showed that the Goa gap (15.8° N) in the Western Ghats is not physical, but a significant vegetational barrier that prevents *Draco dussumieri* from spanning it. In fact, terrestrial reptiles such as skinks (*Ristella beddomei*), King cobra (*Ophiophagus kalinga*) and the Hump‐nosed pit‐viper ( *Hypnale hypnale* ) do not span the Goa Gap either (Chaitanya and Meiri 2022). Similarly, *Dravidogecko* geckos and *Uraeotyphlus* caecilians from the south, and *Thrigmopoeus* tarantulas from the north, abruptly stop in their respective distributions in the Central Western Ghats (Chaitanya et al. 2019; Biswas, unpublished data). There are no significant physical barriers in these regions either that may prevent further dispersal of these organisms, yet little is known about their ecological limits that likely govern their spatial distributions in these continuous environments.

Finally, several systematic studies conducted on Western Ghats endemic taxa, especially on frogs (Van Bocxlaer et al. 2012; Biju et al. 2011; Biju, Garg, Gururaja, et al. 2014; Biju, Garg, Mahony, et al. 2014; Vijayakumar et al. 2014 etc.) and squamates (Chaitanya et al. 2019; Agarwal et al. 2019; Mallik et al. 2020; Mallik et al. 2021 etc.) may have confounded strong mitochondrial divergence across geographic gaps with speciation. These studies have named several new species to capture mitochondrial variation, likely inflating species numbers in these groups. Consequently, the influence of geographic barriers in the Western Ghats in shaping diversity and patterns of diversification may have been overestimated. We advocate the use of genome‐wide datasets coupled with state‐of‐the‐art analytical techniques that include careful testing of potential contact zones to develop a more nuanced understanding of the complex processes that have shaped diversity and distributions in this remarkable landscape.

## Author Contributions

R.C. conducted sampling, analysed data, designed and conducted research, and wrote the manuscript. A.D. performed lab work and conducted research. A.K. conducted sampling, analysed data, and reviewed the manuscript. C.M. conducted sampling, analysed the data and reviewed the manuscript. S.M. reviewed and revised the manuscript, directed and oversaw the research. P.K. generated funding for sequencing, oversaw the wet laboratory work, directed and oversaw the research, reviewed, and revised the manuscript.

## Conflicts of Interest

The authors declare no conflicts of interest.

## Supporting information


Figure S1.

Figure S2.

Figure S3.

Figure S4.

Figure S5.

Figure S6.

Figure S7.

Figure S8.

File S1.

File S2.

File S3.

Data S1.

## Data Availability

DNA alignments, phylogenetic trees, raw Illumina reads, coordinates for sampling localities, occurrence data used for niche modelling and all R scripts used for analyses are archived in the Zenodo digital repository (https://doi.org/10.5281/zenodo.13771314).
