# Supplementary figures and images for: Speciation in the Peninsular Indian Flying Lizard (Draco dussumieri) Follows Climatic Transition and Not Physical Barriers
Source: Mol Ecol. 2025 May 20;34(12):e17800. doi: 10.1111/mec.17800 (PMC12143371; doi:10.1111/mec.17800)

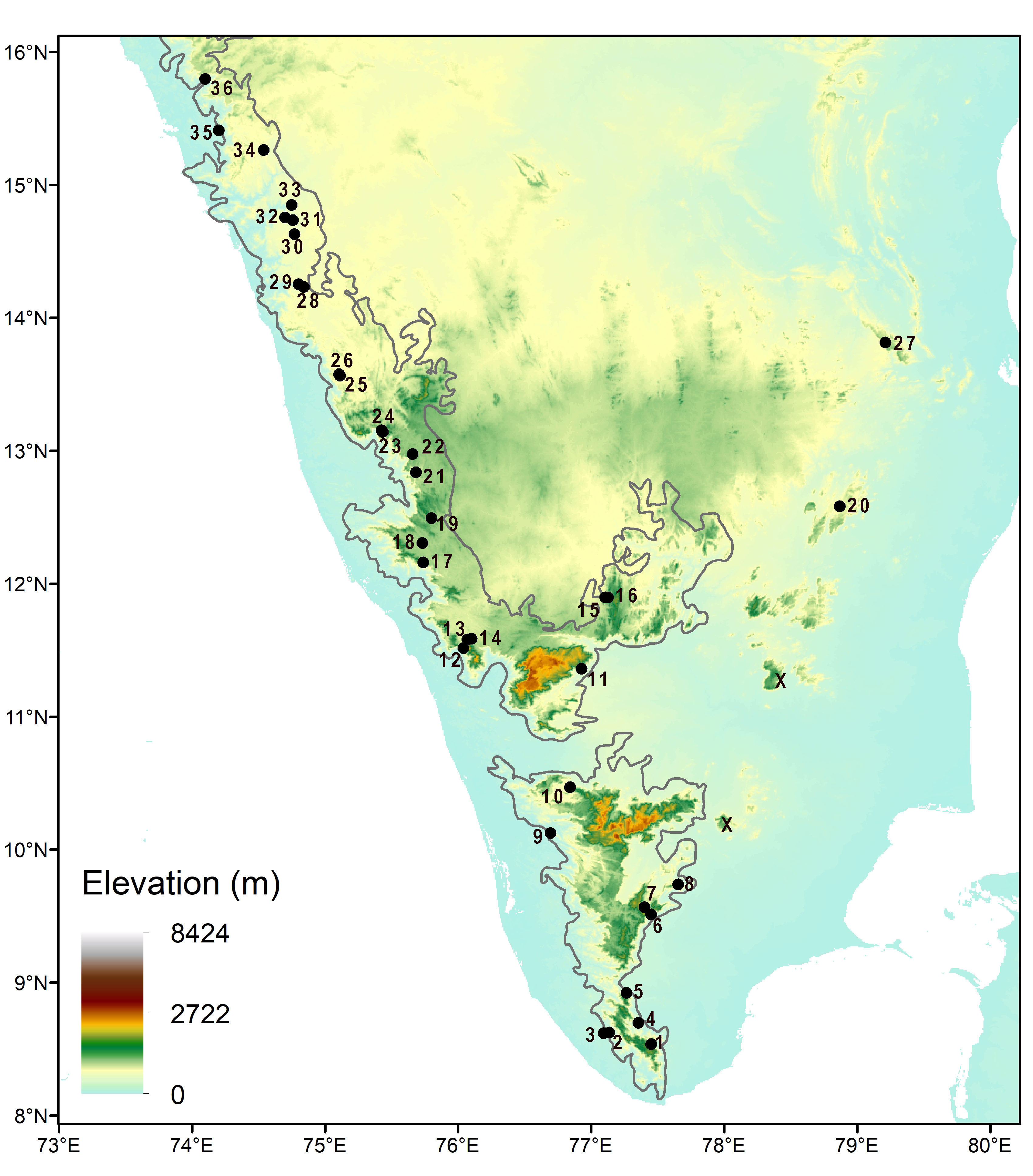

Supplement: Supplementary file 1 — Figure S1. [file MEC-34-e17800-s011.jpg]

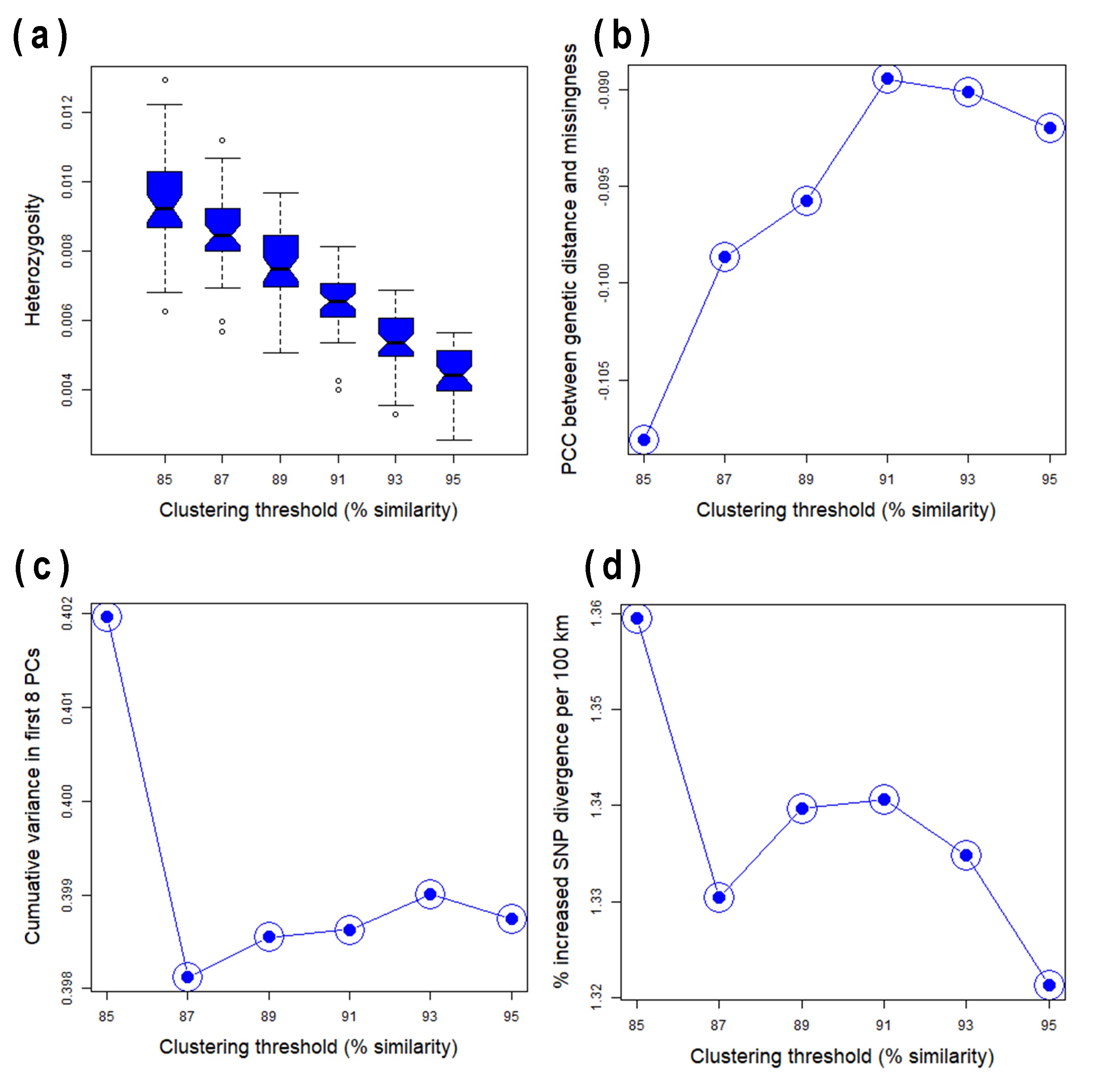

Supplement: Supplementary file 2 — Figure S2. [file MEC-34-e17800-s012.jpg]

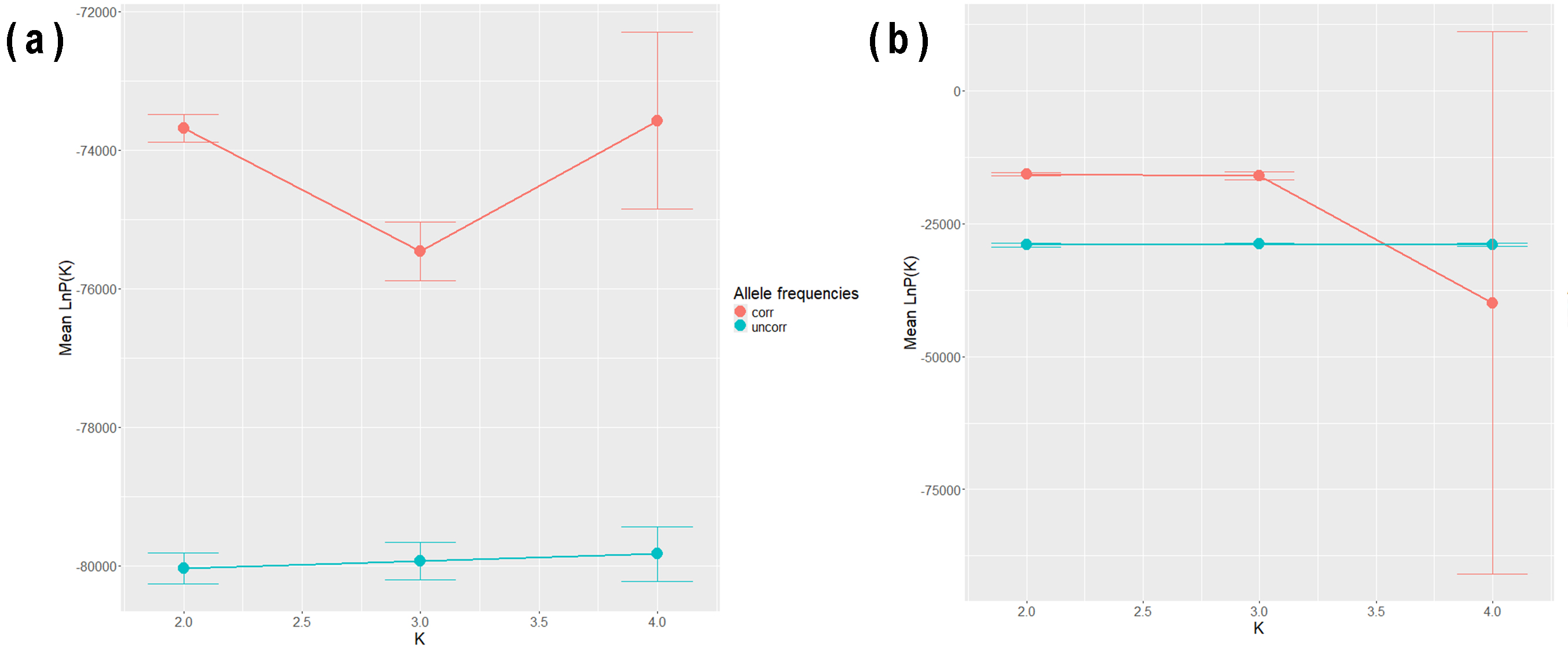

Supplement: Supplementary file 3 — Figure S3. [file MEC-34-e17800-s007.jpg]

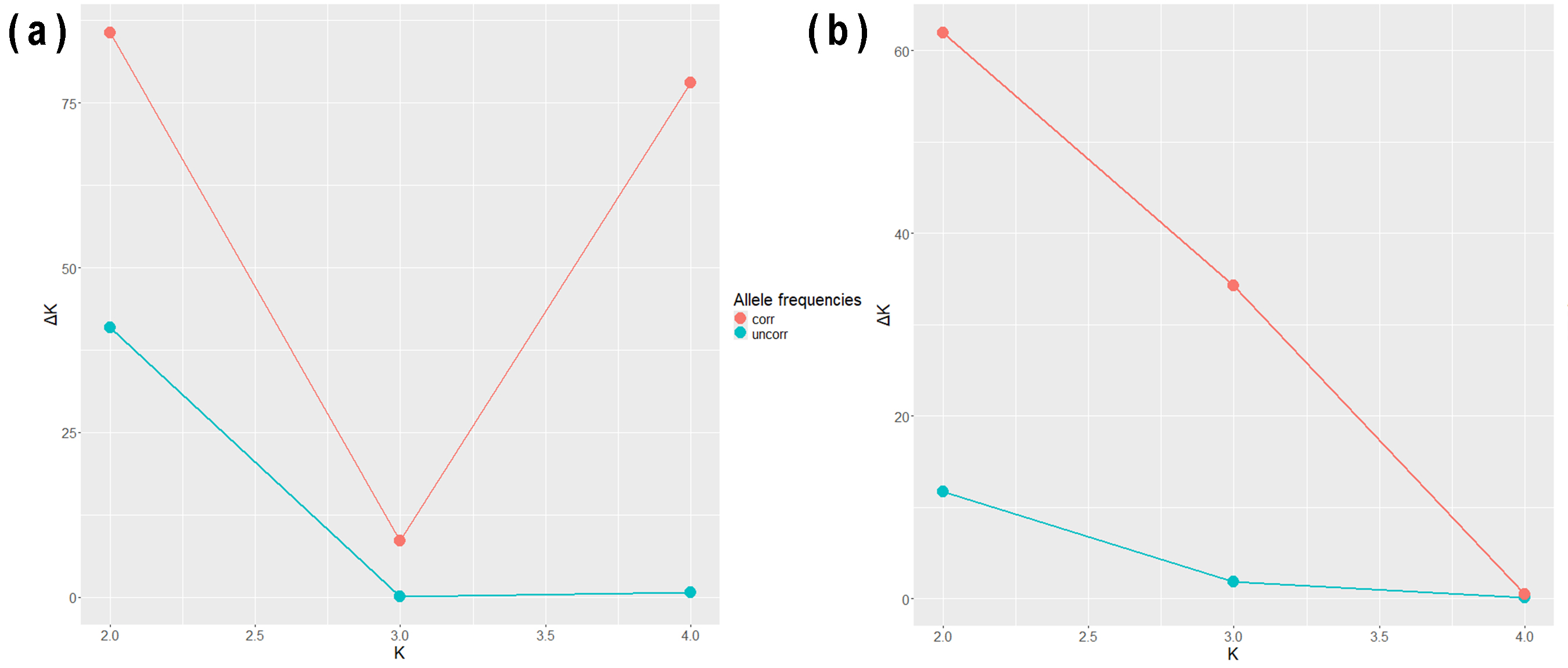

Supplement: Supplementary file 4 — Figure S4. [file MEC-34-e17800-s006.jpg]

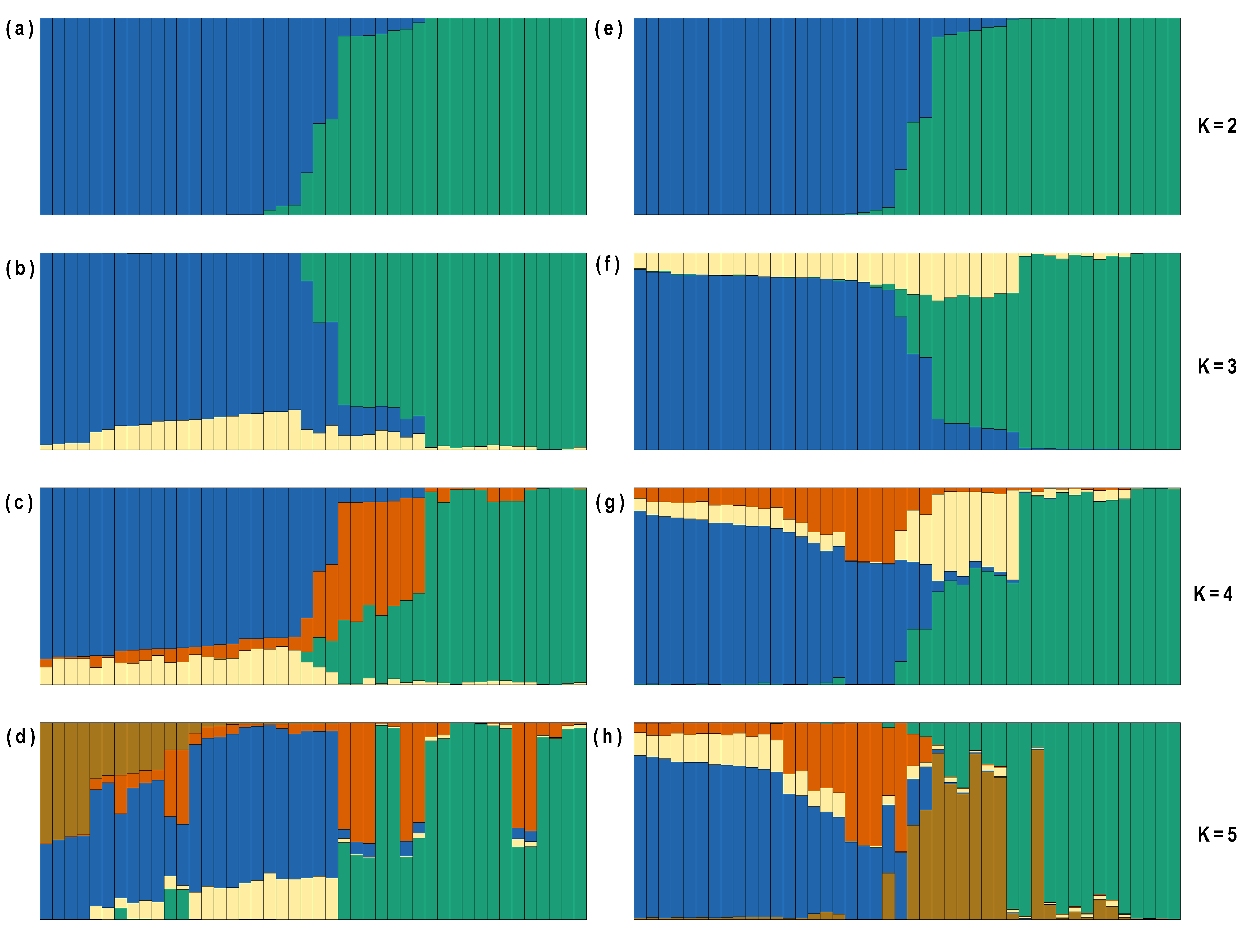

Supplement: Supplementary file 5 — Figure S5. [file MEC-34-e17800-s008.jpg]

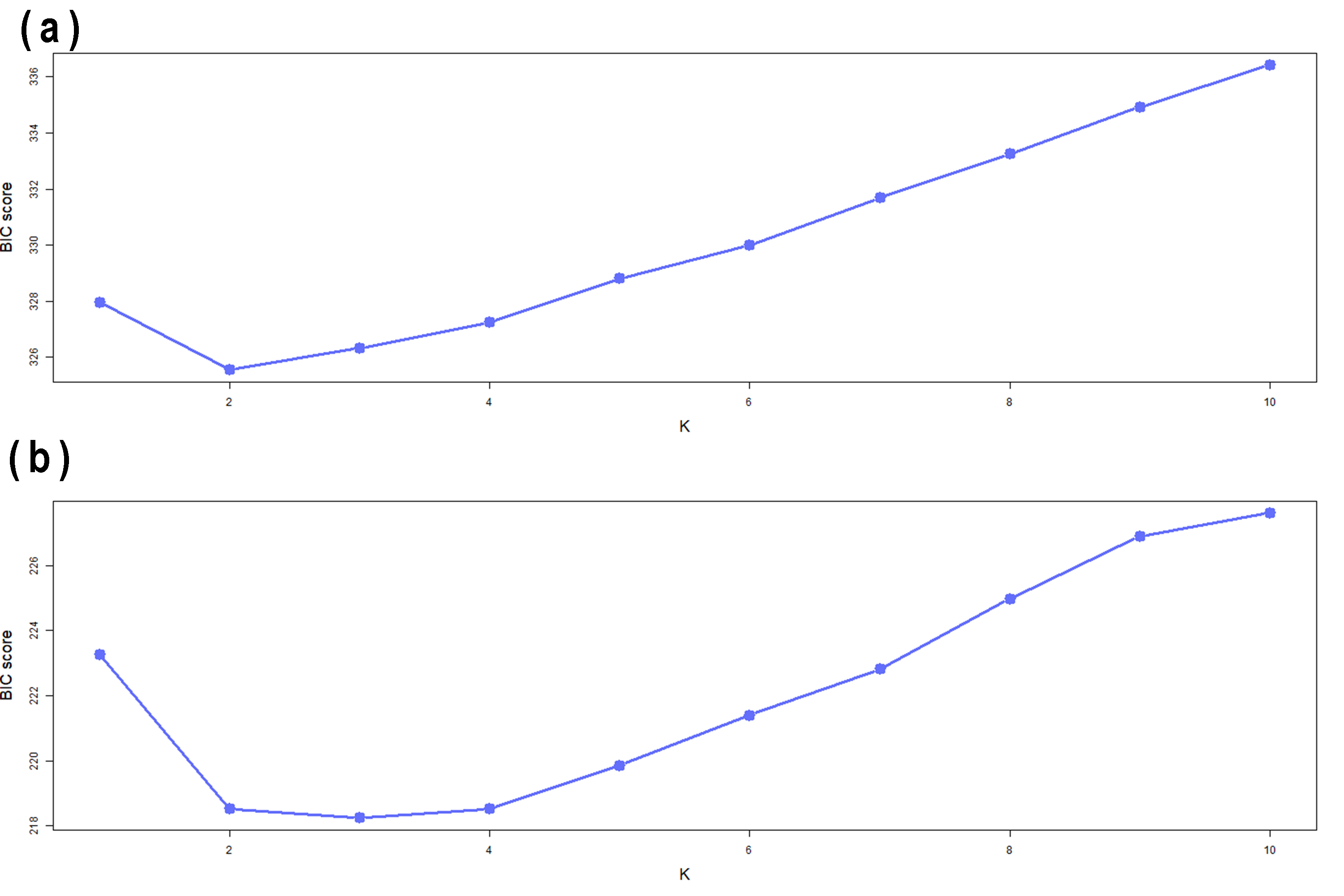

Supplement: Supplementary file 6 — Figure S6. [file MEC-34-e17800-s004.jpg]

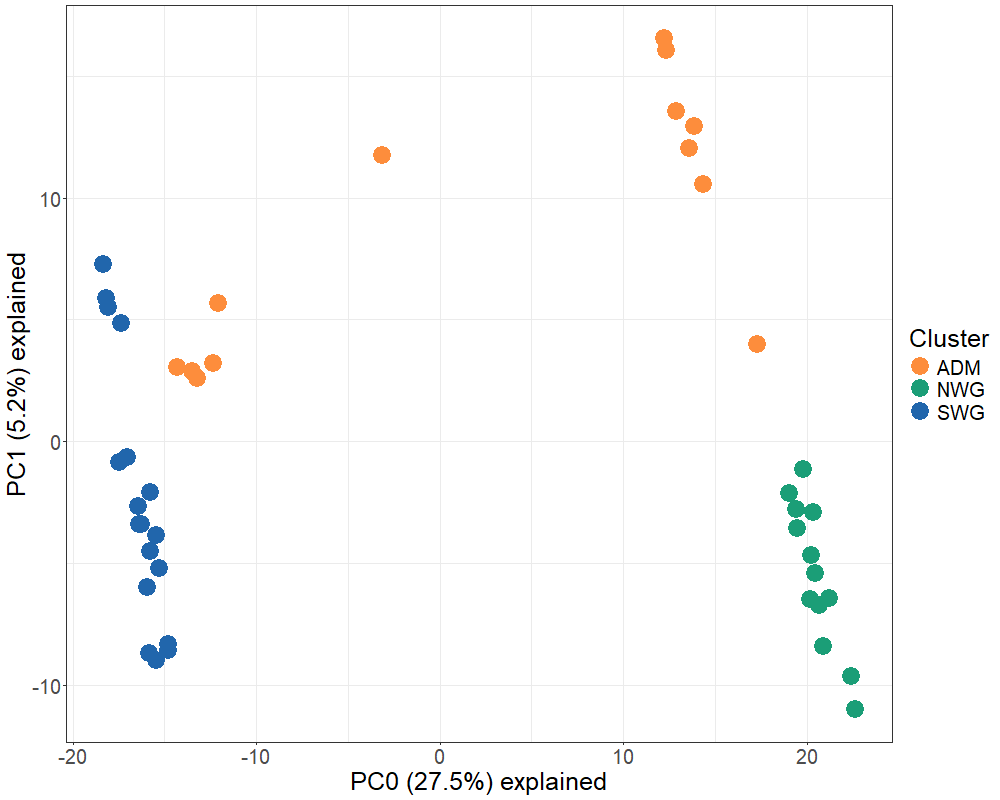

Supplement: Supplementary file 7 — Figure S7. [file MEC-34-e17800-s010.tiff]

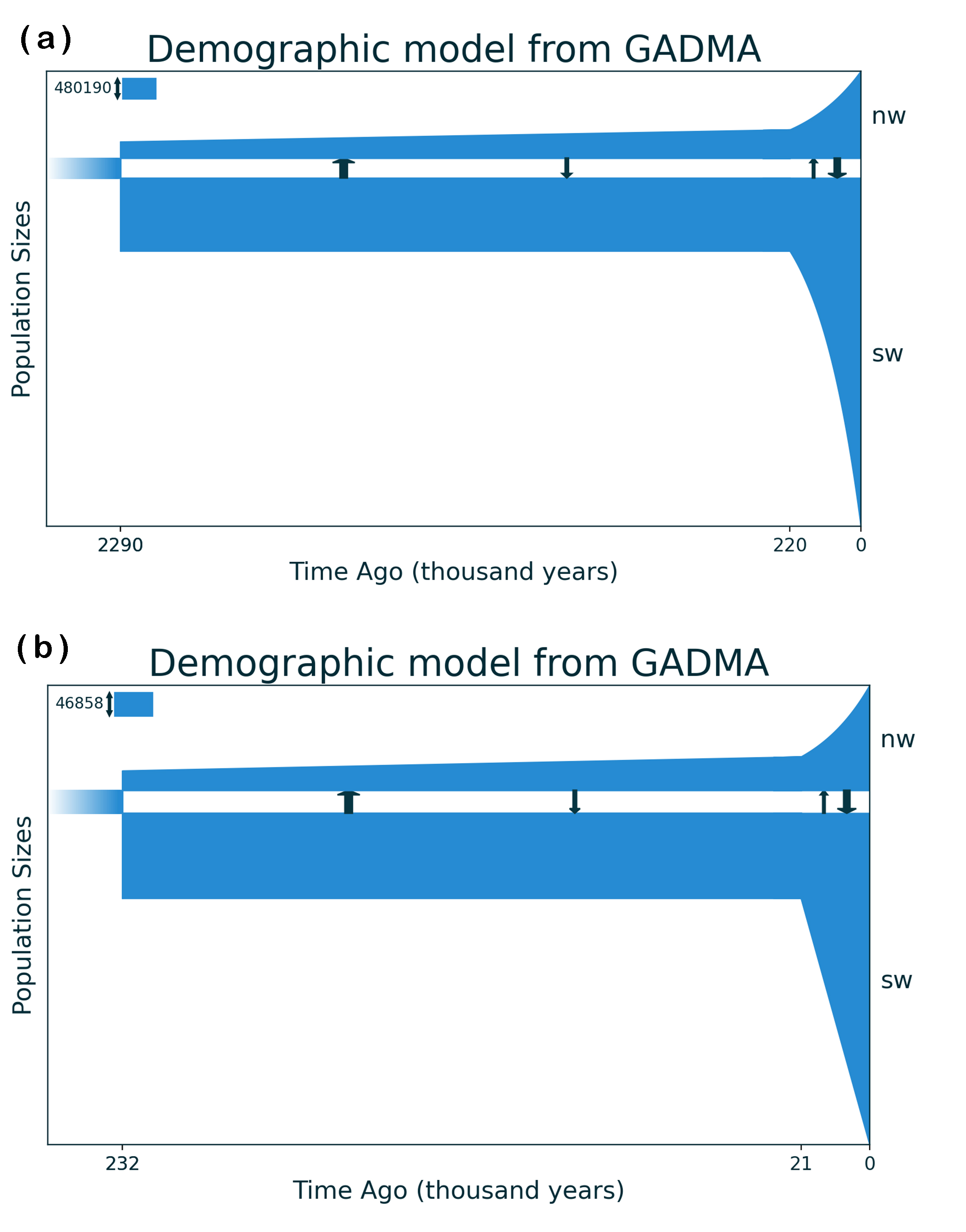

Supplement: Supplementary file 8 — Figure S8. [file MEC-34-e17800-s005.jpg]
