# Supplementary material for: Speciation in the Peninsular Indian Flying Lizard (Draco dussumieri) Follows Climatic Transition and Not Physical Barriers
Source: Mol Ecol. 2025 May 20;34(12):e17800. doi: 10.1111/mec.17800 (PMC12143371; doi:10.1111/mec.17800)
Supplement: Supplementary file 12 — Data S1. [file MEC-34-e17800-s002.docx]

**SUPPLEMENTARY MATERIAL**

**S1. METHODS**

**Methods S1.1: Assembling Sanger-sequenced mitochondrial DNA**

Low quality sequences were edited and trimmed from chromatograms at the 5' and 3' ends by visually inspecting them using the software Chromas v2.6.6 (<http://technelysium.com.au/wp/chromas/>). Sequences were then inspected for mismatched sites by comparing them with their corresponding reverse sequences. The cleaned sequence datasets were then aligned using the MUSCLE algorithm implemented in the program MEGA 11 (Tamura et al., 2021). Alignments of protein-coding DNA sequences were translated to amino acids to detect pseudogenes or premature stop codons that are indicative of frame shifts in the alignment.

**Methods S1.2: Library preparation and Illumina sequencing**

First, we performed a GBS pre-design experiment. The enzymes and sizes of restriction fragments were evaluated using training data. Three criteria were considered: i) The number of tags must be suitable for the specific needs of the research project. ii) The enzymatic tags must be evenly distributed through the sequences to be examined. iii) Repeated tags must be avoided. These considerations improved the efficiency of GBS. To maintain the sequence depth uniformity of different fragments, a tight length range was selected (about 50 bp). Next, we constructed the GBS library in accordance using the pre-designed scheme. Genomic DNA was incubated at 37 ℃ with MseI (New England Biolabs, NEB), T4 DNA ligase (NEB), ATP (NEB), and MseI Y adapter N containing barcode. Restriction-ligation reactions were heat-inactivated at 65 ℃, and then digested for additional restriction enzyme NlaIII(NEB) and EcoRI（NEB） at 37℃. The restriction ligation samples were purified with Agencourt AMPure XP（Beckman）. Then performed PCR reaction using purified samples, Phusion Master Mix (NEB) universal primer and index primer to add index, complete i5 and i7 sequence. The PCR productions were purified using Agencourt AMPure XP (Beckman) and pooled, then run out on a 2% agarose gel. Fragments with 350-400bp (with indexes and adaptors) in size were isolated using a Gel Extraction Kit (Qiagen). These fragment products were then purified using Agencourt AMPure XP (Beckman), which was diluted for sequencing. Pair-end sequencing was then performed upon the selected tags using an Novaseq6000 high-throughput sequencing platform followed by SNP genotyping and evaluation.

**Methods S1.3: Details of the MaxEnt modelling conducted on the parental and admixed lineages**

We constructed maximum entropy climatic niche models for the northern, southern and admixed lineages using MaxEnt version 3.4.3 (Phillips et al., 2006). MaxEnt is a learning method that incorporates presence-only data and environmental variables to define and delimit the distribution of the maximum entropy. We chose MaxEnt since the underlying algorithm is less likely to be influenced by small sample sizes (Kumar & Stohlgren, 2009; Pearson et al., 2007) and uses a regularization parameter (β**)** to ensure models are not over-fitted (Phillips et al., 2006). To test for niche divergence between the parental northern and southern lineages, and the impacts of LGM climate change on them, occurrence data for these lineages were used separately to model Anthropocene distributions and projected onto the LGM dataset. Georeferenced data were randomly partitioned to allow 90% training data to fit models and 10% to validate model predictions. Thirty replicates of each model were generated by bootstrapping to estimate variability, and the maximum number of background points was set to 10,000. Bootstrapping was preferred over the cross-validation method due to the small presence-only datasets. We selected both linear and step functions (linear, quadratic, product, and threshold) features classes and allowed the MaxEnt ‘auto features’ option to automate the task of choosing the right feature types based on our sample sizes. We tested models calibrated with different values for β (0.25, 0.5,1.0, 2.0 and 5.0: 1.0 is the default) using the auto features option and used each model's discriminatory ability (AUC) and omission rates to choose the regularization multiplier that is best suited for our analyses. All models constructed using β ≤ 1.0 resulted in models that were only marginally different, with low omission rates and high discriminatory ability, and therefore β = 1.0 (the Maxent default) was selected for the rest of our analyses. The jackknife test in MaxEnt was used to estimate the contribution and significance of each variable to the final model when used in isolation, as well as when omitted from the composite set of variables used to determine the model. The mean (of 30 replicate runs) suitability maps generated by the logistic output format were obtained that depict an estimated probability of presence for the geographic extent, with values ranging from 0 (unsuitable) to 1 (suitable). We used the threshold-independent measure, Area under the receiver operating characteristic curve (AUC), to evaluate model performance. AUC calculates the ability of a model to discern areas where the focal species occurs and areas where it does not (1: perfect predictive ability, 0: no predictive ability) and we used a value of AUC>0.9 to determine strong model performance. For further validation, we used the true skill statistic (TSS) which is a threshold-dependent measure that accounts for model omission and commission errors (Allouche et al., 2006). The method takes as input the predicted ecological model and georeferenced presence/absence data, and uses Cohen’s kappa (*κ*) which corrects the accuracy of model prediction by the accuracy expected to occur by chance. For each lineage, we used the presence points of the other two lineages as absence data. For instance, the occurrence points of NWG and ADM were used as absence data to evaluate models constructed for SWG. Cohen’s kappa (*κ*) ranges from −1 to +1, where a value of +1 indicates a perfectly non-random prediction and values of 0 or less indicate performance no better than random (Allouche et al., 2006). The TSS analysis was carried out in the R package *ntbox* (Osorio-Olvera et al., 2020) and its own optimal threshold was used to evaluate model performance. Model performances based on TSS were scored following Araújo et al. (2005): excellent if *κ* > 0.75, good if 0.75 > *κ* > 0.4 and poor if *κ* < 0.4. Models were considered useful if TSS ranged from 0.75 to 0.5. The two model-performance metrics were applied on the present-day models constructed for all three lineages.

**S2. TABLES**

**Table S1:** Markers used for phylogenetic reconstructions in this study

| **Locus** | **Primers** | **Sequences** | **Sequence length** | **Reference** |
| --- | --- | --- | --- | --- |
| ND2 | Metf.1  ALAr.2m* | 5’-AAGCAGTTGGGCCCATRCC-3’  5’-AAAGTGTCTGAGTTGCATTCRG-3’ | 1041 | Macey et al., 1997 |
| 12S | H1478  L10091 | 5’-GAGGGTGACGGGCGGTGTGT-3’  5’-AAACTGGGATTAGATACCCCACTAT-3’ | 502 | Kocher et al., 1989 |
| 16S | L2606  H3056 | 5’-CTGACCGTGCAAAGGTAGCGTAATCACT-3’  5’-CTCCGGTCTGAACTCAGATCACGTAGG-3’ | 456 | Hass et al., 1993 |

*Primer modified from Macey et al. (1997). See McGuire and Kiew (2001).

**Table S2:** Details of the genomic datasets used in this study and the analyses they were used for.

| **ddRAD dataset** | **Outgroups included** | **Min # individuals/locus** | **# of loci** | **# variable sites** | **# parsimony informative sites** | **# of unlinked SNPs** | **Analyses** |
| --- | --- | --- | --- | --- | --- | --- | --- |
| min04 | No | 4 | 277889 | 1571099 | 719719 | 265509 | Cluster threshold for ipyrad assembly |
| min22 | No | 22 | 27971 | 231730 | 125283 | 27858 | STRUCTURE, find.clusters, PCA |
| min33 | No | 33 | 2204 | 22630 | 12794 | 2197 | STRUCTURE, find.clusters, PCA, triangulaR, GADMA, IBD, GDM, RDA |

**Table S3:** Evaluation metrics for the MaxEnt models generated for the three populations of D. dussumieri

| **Population** | **AUC** | **TSS** |
| --- | --- | --- |
| Northern | 0.974±0.005 | 0.951 |
| Southern | 0.946±0.008 | 0.940 |

**S3. FIGURES**

**Figure S1:** Elevation map of Peninsular India showing localities sampled during this study and numbered latitudinally. Points marked ‘X’ are localities where Draco dussumieri were found, but not sampled. Details of the localities (by locality number) are listed in Supplementary file S4.1

**Figure S2:** The four metrics used in this study to evaluate the optimum clustering threshold to assemble our ddRAD data. (a) per-individual percent heterozygosity (b) Pearson’s correlation coefficient between pairwise genetic dissimilarity and data missingness (c) cumulative variance explained by the first eight principal components retained from a principal component analysis (d) percentage increase in SNP divergence per 100 km (isolation by distance)

**Figure S3:** Mean log-likelihood values for the number of inferred population clusters (K) using the (a) min22 and (b) min33 datasets.

**Figure S4:** The *∆K* statistic estimated for the number of inferred population clusters (K) using the (a) min22 and (b) min33 datasets.

**Figure S5:** Bar plot results of the STRUCTURE analyses for the number of inferred population clusters (K) using a model of correlated frequencies conducted on the min22 (a,b,c,d) and the min33 (e,f,g,h) datasets. The arrangement of individuals in each bar plot is roughly according to latitude (south to north).

**Figure S6:** K-means tests conducted using the find.clusters() function on the (a) min22 and (b) min33 datasets, by varying K from 1 to 10.

**Figure S7:** The first two Principal components averaged from 25 replicate PCAs conducted on the min22 dataset showing genetic segregation of the parental and admixed lineages.

**Figure S8:** Demographic models constructed in GADMA using (a) four time-intervals after initial divergence and (b) using the fastest nuclear mutation rate available in literature (1x10^-8^ substitutions/site/year).

**S.4 Files**

**File S1:** Details of the number of individuals, number of mtDNA sequences, and number of DDRad sequences generated at each sampling locality (see Figure S1) for this study.

**File S2:** Details of the climatic niche models conducted using MaxENT for the northern lineage (NWG) during the present and the LGM.

**File S3: –** Details of the climatic niche models conducted using MaxENT for the southern lineage (SWG) during the present and the LGM.

**REFERENCES**

Allouche, O., Tsoar, A., & Kadmon, R. (2006). Assessing the accuracy of species distribution models: Prevalence, kappa and the true skill statistic (TSS). *Journal of Applied Ecology*, *3*(6), 1223–1232.

Hass, C.A., Hedges, S.B., Maxson, L.R. (1993). Molecular insights into the relationships and biogeography of West Indian Anoline lizards. *Biochemical Systematics and Ecology*. 21(1):97-114. DOI: 10.1016/0305-1978(93)90015-J

Kocher T.D., Thomas W.K., Meyer A., Edwards S.V., Pääbo S., et al. (1989). Dynamics of mitochondrial DNA evolution in animals: amplification and sequencing with conserved primers. *PNAS*. 86:6196– 6200. DOI: 10.1073/pnas/86.16.6196

Kumar, S., & Stohlgren, T. J. (2009). Maxent modeling for predicting suitable habitat for threatened and endangered tree *Canacomyrica monticola* in New Caledonia. *Journal of Ecology and Natural Environment*, *1*, 94–98.

Macey, J.R., Schulte II, J.A., Larson, A., Fang, Z., Wang, Y., Tuniyev, B.S., Papenfuss, T.J. (1998). Phylogenetic Relationships of Toads in the Bufo bufo Species Group from the Eastern Escarpment of the Tibetan Plateau: A Case of Vicariance and Dispersal. *Molecular Phylogenetics and Evolution*. 9(1):80-87. DOI: 10.1006/mpev.1997.0440

McGuire, J.A. and Heang, K.B., 2001. Phylogenetic systematics of Southeast Asian flying lizards (Iguania: Agamidae: Draco) as inferred from mitochondrial DNA sequence data. *Biological Journal of the Linnean Society*, *72*(2), pp.203-229.

Pearson, R. G., Raxworthy, C. J., Nakamura, M., & Peterson, A. T. (2007). Predicting species distributions from small numbers of occurrence records: A test case using cryptic geckos in Madagascar. *Journal of Biogeography*, *34*, 102–117.

Phillips, S. J., Anderson, R. P., & Schapire, R. E. (2006). Maximum entropy modeling of species geographic distributions. *Ecological Modelling*, *190*, 231–259.

Osorio-Olvera, L., Lira-Noriega, A., Soberón, J., Townsend Peterson, A., Falconi, M., Contreras-Díaz, R. G., Martínez-Meyer, E., Barve, V., & Barve, N. (2020). ntbox: An R package with graphical user interface for modeling and evaluating multidimensional ecological niches. *Methods in Ecology and Evolution*, *11*, 1199–1206. <https://doi.org/10.1111/2041-210X.13452>

Tamura, K., Stecher, G. and Kumar, S., 2021. MEGA11: molecular evolutionary genetics analysis version 11. *Molecular biology and evolution*, *38*(7), pp.3022-3027.
